# Supplementary material for: Identification and Relative Quantification of hFSH Glycoforms in Women’s Sera via MS–PRM-Based Approach
Source: Pharmaceutics. 2021 May 27;13(6):798. doi: 10.3390/pharmaceutics13060798 (PMC8226871; doi:10.3390/pharmaceutics13060798)
Supplement: Supplementary file 1 [file pharmaceutics-13-00798-s001.zip › pharmaceutics-1156624-supplementary.pdf]

pharmaceutics

Supplementary Materials: Identification and Relative Quantification of hFSH Glycoforms in Women's Sera via MS–PRM-Based Approach

Chiara Melchiorre, Cerina Chhuon, Vincent Jung, Joanna Lipecka, Francesca Di Rella, Alessandro Conforti, Angela Amoresano, Andrea Carpentieri and Ida Chiara Guerrera

Immunoprecipitation recovery

In order to calculate the recovery and reproducibility of the immunoprecipitation (IP), 3 new pooled sera aliquots were subjected to IP and PRM analysis. We started from 3 × 250 µL of sera pool (see material and methods) diluted 5 times. The 3 aliquots were then spiked with 1ng/µL of FSH standard and 2,5 µL of each spiked serum sample were stored (Input_spike). The spiking was necessary to detect the FSH in the Input samples. Immunoprecipitation protocol (see material and methods) was performed on each spiked sample and the flow-through was stored as negative control. We then ran three replicated IP_spiked experiments, and we monitored the non-glycosylated peptide of FSH (ETVRVPGCAHHADSLY, m/z = 906.4309, z = 2) before IP (Input), in the flow-through of the IP (FT) and eluates (IP). The scheme of the sample preparation is reported in Figure S1:

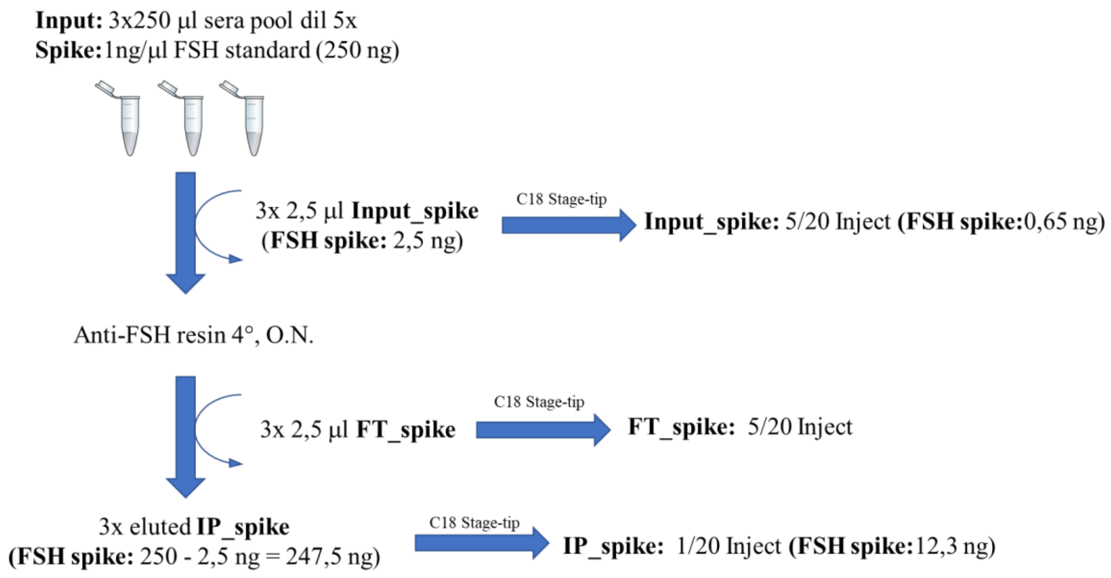

Figure S1. Workflow of samples preparation for IP recovery calculation.

Data obtained from PRM MS analyses (triplicates) led to the identification of FSH in both sample (Input and IP). FSH was never detect in the FT suggesting a reproducible high yield if the immunoprecipitation. The recovery was calculated by measuring the best transition area of peptide ETVRVPGCAHHADSLY (906.4309 m/z, z = 2) in both IP_spike and Input_spike. As an example in the figure below we report selected transition of A) Input spiked sample; B) IP spiked sample; C) Flow through spiked sample. Figure S2 shows the results about recovery calculated on the Quantifier (best transition: b10-1107.5364) averaged Area before and after IP. The average area of IP_spike was calculated taking into consideration the dilutions and the injected quantity (injected FSH quantity in IP_spike was about 20-fold higher than Input one).

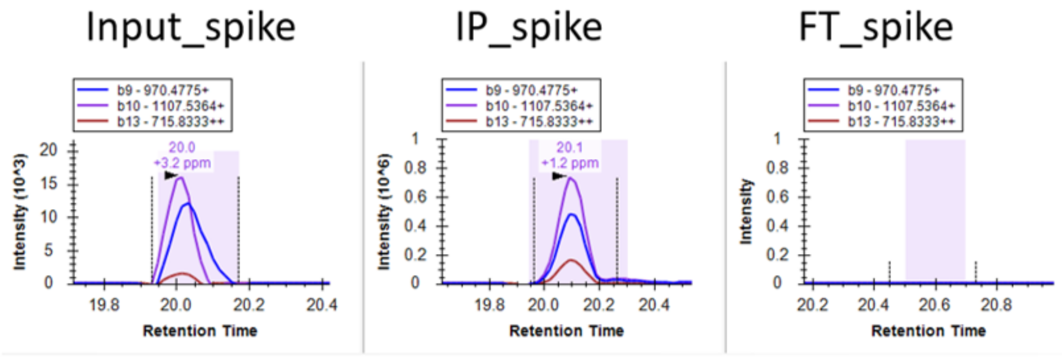

β-FSH peptide :ETVRVPGCAHHADSLY
Precursor m/z : 906.4309 (2+)
Elution time 20.00
CE 35

PRM experiments:	Peptide: ETVRVPGCAHHADSLY		m/z = 906.4309	z = 2
SAMPLES	Quantifier AREA (Average)		Recovery: 89%	
Input_spiked samples		77273		
IP_spiked samples	1379557 (20×)	68978		

Figure S2. Skyline chromatographic traces post-acquisition extraction the of best 3 selected fragment ion from peptide ETVRVPGCAHHADSLY (906.4309 m/z, z = 2) yield before (Input_spike), after the immuprecipitation (IP_spike) and in the unbound (FT_spike). The best transition 906.43092+→1107+ is selected as quantifier. The area in IP_Spike is calculated taking into consideration the dilutions and the injected quantity.

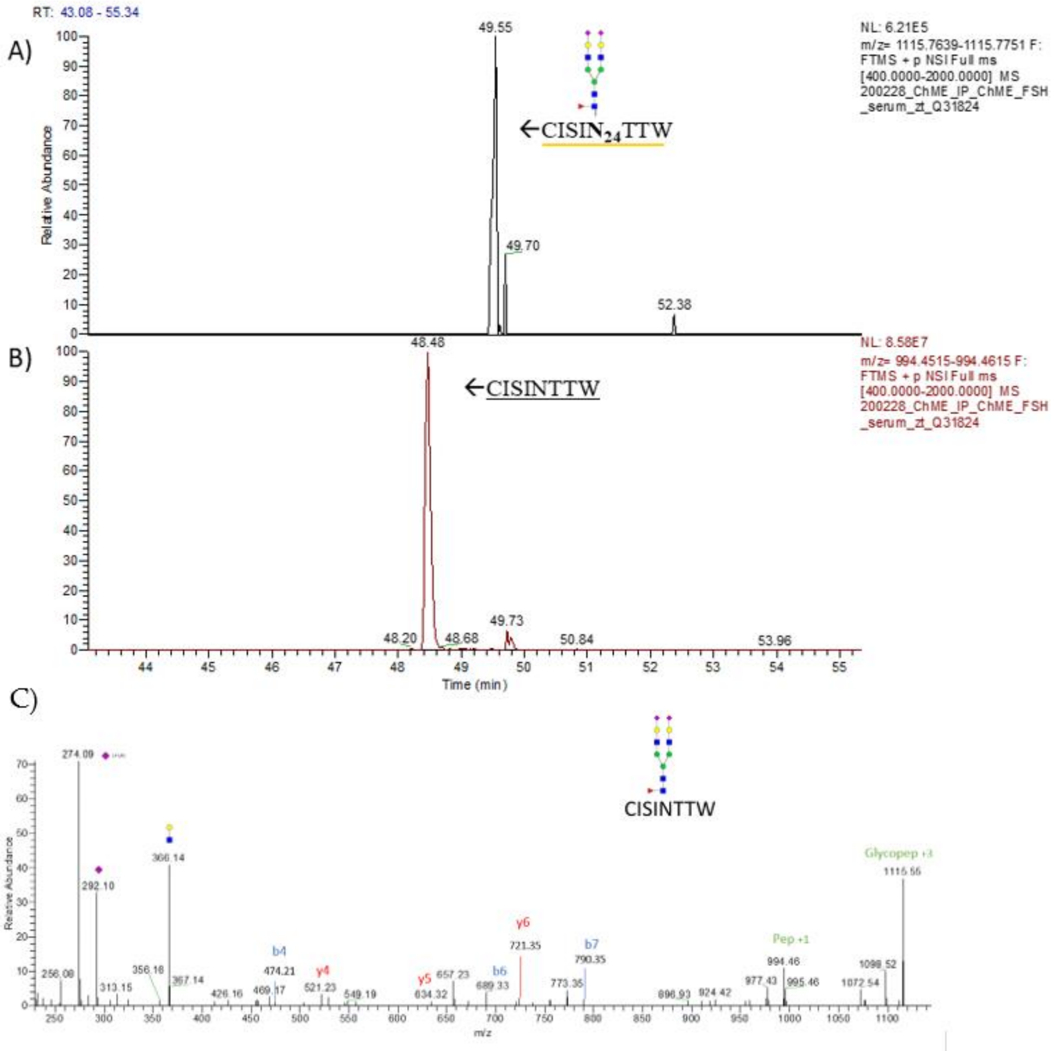

Figure S3. LC-MS/MS full scan analysis of serum IP_hFSH. Extracted-ion chromatogram (XIC) comparison between: (**A**) glycopeptide CISIN*TTW m/z = 1115,77 (RT:49.55 min; NL: 6.21E5), (**B**) unmodified one m/z = 994,46 (RT: 48.48 min; NL: 8.58E7) and (**C**) glycopeptide CISIN*TTW m/z = 1115,77 MS2 spectrum.

Table S1. Full LC-MS/MS identification of RHS_FSH standard. Mass spectrometry data were analysed by Proteome Discoverer v2.4 (Thermo Scientific, Waltham, MA, USA) against human subset from UniProtKB/Swiss-Prot complete proteome database using the Byonic node search engine (v3.6.0 Protein Metrics, PMI-Suite). Each identified glycopeptides were further validate by interpreting MS/MS spectrum. In bold is higlithed the glycopeptides used for the validation example showed in Figure 2.

N-Glycan site	Peptide	Glycans composition	Short Name	Observed m/z	z	Observed (M+H)	Calc. Mass (M+H)	Scan Time (min)	Note
N78	F.KVEN[+1913.677]HTACHCSTCY.Y	HexNAc(4)Hex(5)NeuAc(1)	A2G2S1	1227.465	3	3680.381	3680.381	14.52	Validate by MS/MS
N78	F.KVEN[+2204.772]HTACHCSTCY.Y	HexNAc(4)Hex(5)NeuAc(2)	A2G2S2	993.625	4	3971.478	3971.477	15.24	Validate by MS/MS
N78	F.KVEN[+2569.905]HTACHCSTCY.Y	HexNAc(5)Hex(6)NeuAc(2)	A3G3S2	1084.907	4	4336.608	4336.609	15.24	Validate by MS/MS
N52	L.VQKN[+1913.677]VTSESTCCVAKSY.N	HexNAc(4)Hex(5)NeuAc(1)	A2G2S1	969.402	4	3874.585	3874.587	20.17	Validate by MS/MS
N52	L.VQKN[+1458.442]VTSESTCCVAKSY.N	HexNAc(5)Hex(6)NeuAc(3)	A3G3S3	1140.455	3	3419.352	3419.352	20.27	
**N52**	**L.RSKKTMLVQKN[+2204.772]VTSESTCCVAKSY.N**	**HexNAc(4)Hex(5)NeuAc(2)**	**A2G2S2**	**1253.301**	**4**	**5010.175**	**5010.178**	**20.28**	**Validate by MS/MS**
N52	L.RSKKTMLVQKN[+1458.442]VTSESTCCVAKSY.N	HexNAc(2)Hex(6)Phospho(1)	Man6_P	853.575	5	4263.846	4263.8476	18.67	Validate by MS/MS
N52	L.VQKN[+2204.772]VTSESTCCVAKSY.N	HexNAc(4)Hex(5)NeuAc(2)	A2G2S2	1389.233	3	4165.684	4165.683	23.38	Validate by MS/MS
	L.QCM[+15.995]GCCF.S			489.651	2	978.294	978.294	23.49	
	Y.NRVTVM[+15.995]GGF.K			498.75	2	996.492	996.493	23.64	
N78	Y.NRVTVMGGFKVEN[+2204.772]HTACHCSTCY.Y	HexNAc(4)Hex(5)NeuAc(2)	A2G2S2	1233.995	4	4932.959	4932.957	25.31	Validate by MS/MS
	L.QCMGCCF.S			481.653	2	962.299	962.299	28.73	
	Y.NRVTVMGGF.K			490.753	2	980.498	980.498	28.97	
	F.SRAYPTPL.R			452.748	2	904.489	904.489	32.75	
	F.FSQPGAPIL.Q			465.258	2	929.509	929.509	37.67	
	F.SQPGAPILQCM[+15.995]GCCF.S			871.362	2	1741.716	1741.717	38.06	
	L.QENPFF.S			781.351	1	781.351	781.352	40.1	
	F.FSQPGAPILQCM[+15.995]GCCF.S			944.894	2	1888.781	1888.785	41.32	
	F.SQPGAPILQCMGCCF.S			863.364	2	1725.72	1725.722	44.47	
	L.QENPF.F			634.283	1	634.283	634.283	44.75	
	Y.KDPARPKIQKTCTFKEL.V			687.382	3	2060.133	2060.132	17.29	
	L.VYKDPARPKIQKTCTF.K			488.766	4	1952.044	1952.042	18.08	
	Y.CYTRDL.V			414.189	2	827.371	827.372	18.65	
	Y.ETVRVPGCAHHADSL.Y			824.897	2	1648.786	1648.786	18.93	
	Y.PVATQCHCGKCDSDSTDCTVRGL.G			656.778	4	2624.091	2624.096	18.94	

	Y.CSFGEMK.E			429.678	2	858.348	858.348	19.76	
	Y.TRDLVYKDPAR-PKIQKTCTF.K			610.081	4	2437.303	2437.302	20.27	
	Y.CSFGEMKE.-			494.199	2	987.391	987.391	21.34	
	Y.KDPARPKIQKTCT-FKELVY.E			774.759	3	2322.264	2322.264	21.62	
	Y.ETVRVPGCAHHADSLY.T			906.428	2	1811.848	1811.849	21.83	
	Y.PVATQCHCGKCDSDSTDC TVRGLGPSY.C			757.821	4	3028.261	3028.265	22.32	
	Y.TYPVATQCHCGKCDSDSTD CTVRGL.G			722.804	4	2888.193	2888.207	22.86	
	Y.TYPVATQCHCGKCDSDSTD CTVRGLGPSY.C			1098.129	3	3292.371	3292.376	25.27	
	L.VYETVRVPG-CAHHADSLY.T			691.998	3	2073.98	2073.981	26.37	
	Y.CYTRDLVY.K			545.256	2	1089.505	1089.503	26.61	
	L.GPSYCSFGEMKE.-			696.283	2	1391.559	1391.561	27.74	
	Y.TYPVATQCHCGKCDSDSTD CTVRGLGPSYCSF.G			922.38	4	3686.499	3686.507	28.95	
	L.GPSYCSF.G			409.163	2	817.318	817.319	30.05	
	L.TNITIAIEKEECRF.C			862.44	2	1723.872	1723.868	31.46	
	Y.KDPARPKIQKTCTF.K			563.974	3	1689.909	1689.911	34.55	
N24	F.CISIN[+1768.640]TTW.C	HexNAc(4)Hex(5)Fuc(1)	FA2G2	1382.053	2	2763.098	2763.106	35.58	Validate by MS/MS
	F.CISINTTW.C			497.737	2	994.466	994.466	39.63	
N24	F.CISIN[+2205.793]TTW.C	HexNAc(4)Hex(5)Fuc(2) NeuAc(1)	F2A2G2S1	1067.425	3	3200.259	3200.259	42.85	Validate by MS/MS
N25	F.C[+57.021]ISIN[+1458.442]TT W.C	HexNAc(2)Hex(6)Phos-pho(1)	Man6_P	1226.957	2	2452.906	2452.9083	45.07	Validate by MS/MS
N24	F.CISIN[+2059.735]TTW.C	HexNAc(4)Hex(5)Fuc(1) NeuAc(1)	FA2G2S1	1527.601	2	3054.195	3054.201	43.11	Validate by MS/MS
N24	F.CISIN[+2076.750]TTW.C	HexNAc(4)Hex(6)Fuc(2)	F2A2G3	1024.413	3	3071.226	3071.217	43.6	Validate by MS/MS
N24	F.CISIN[+1913.677]TTW.C	HexNAc(4)Hex(5)NeuA c(1)	A2G2S1	1454.578	2	2908.148	2908.143	43.64	Validate by MS/MS
N24	F.CISIN[+1930.692]TTW.C	HexNAc(4)Hex(6)Fuc(1)	FA2G3	975.727	3	2925.167	2925.159	43.67	Validate by MS/MS
N24	F.CISIN[+2715.963]TTW.C	HexNAc(5)Hex(6)Fuc(1) NeuAc(2)	FA3G3S2	1237.481	3	3710.428	3710.429	48.88	Validate by MS/MS
N24	F.CISIN[+2643.941]TTW.C	HexNAc(6)Hex(7)NeuA c(1)	A3G4S1	1819.202	2	3637.397	3637.412	49.09	Validate by MS/MS
N24	F.CISIN[+2496.888]TTW.C	HexNAc(4)Hex(5)Fuc(2) NeuAc(2)	F2A2G2S2	1164.455	3	3491.35	3491.355	49.78	Validate by MS/MS

N24	F.CISIN[+2350.830]TTW.C	HexNAc(4)Hex(5)Fuc(1) NeuAc(2)	FA2G2S2	1115.771	3	3345.297	3345.297	49.98	Validate by MS/MS
N24	F.CISIN[+2204.772]TTW.C	HexNAc(4)Hex(5)NeuA c(2)	A2G2S2	1067.085	3	3199.24	3199.239	49.99	Validate by MS/MS
N24	F.CISIN[+1298.476]TTW.C	HexNAc(4)Hex(3)	A2G0	1146.974	2	2292.941	2292.942	43.41	Validate by MS/MS
N7	L.TN[+2715.962]ITIAIE-KEECRF.C	HexNAc(5)Hex(6)Fuc(1) NeuAc(2)	FA3G3S2	1480.89	3	4440.67	4439.83	29.44	
N7	L.TN[+2465.894]ITIAIE-KEECRF.C	HexNAc(6)Hex(5)Fuc(1) NeuAc(1)	FA4G2S1	1397.27	3	4189.81	4189.69	26.37	
N7	L.TN[+2204.772]ITIAIE-KEECRF.C	HexNAc(4)Hex(5)NeuA c(2)	A2G2S2	1310.17	3	3928.51	3928.63	17.41	
N7	L.TN[+2350.830]ITIAIE-KEECRF.C	HexNAc(4)Hex(5)Fuc(1) NeuAc(2)	FA2G2S2	1358.9	3	4074.7	4074.7	22.91	

Table S2. LC-MS/MS identification of IP_hFSH serum sample. Mass spectrometry data were analysed by Proteome Discoverer v2.4 (Thermo Scientific) against human subset from UniProtKB/Swiss-Prot complete proteome database using the Byonic node search engine (v3.6.0 Protein Metrics. PMI-Suite).

Accession Number	Protein Description	# Peptides	# PSMs	# Unique Peptides	# AAs	MW [kDa]
P08603	Complement factor H OS=Homo sapiens OX=9606 GN=CFH PE=1 SV=4	114	312	116	1231	139
P02768	Serum albumin OS=Homo sapiens OX=9606 GN=ALB PE=1 SV=2	78	302	96	609	69.3
P0DOX5	Immunoglobulin gamma-1 heavy chain OS=Homo sapiens OX=9606 PE=1 SV=2	44	295	46	449	49.3
P01871	Immunoglobulin heavy constant mu OS=Homo sapiens OX=9606 GN=IGHM PE=1 SV=4	52	240	53	453	49.4
P0DOX7	Immunoglobulin kappa light chain OS=Homo sapiens OX=9606 PE=1 SV=1	25	125	25	214	23.4
P01023	Alpha-2-macroglobulin OS=Homo sapiens OX=9606 GN=A2M PE=1 SV=3	55	106	62	1474	163.2
P01859	Immunoglobulin heavy constant gamma 2 OS=Homo sapiens OX=9606 GN=IGHG2 PE=1 SV=2	15	98	16	326	35.9
P01876	Immunoglobulin heavy constant alpha 1 OS=Homo sapiens OX=9606 GN=IGHA1 PE=1 SV=2	37	98	37	353	37.6
P02787	Serotransferrin OS=Homo sapiens OX=9606 GN=TF PE=1 SV=3	41	97	47	698	77
P07358	Complement component C8 beta chain OS=Homo sapiens OX=9606 GN=C8B PE=1 SV=3	45	90	45	591	67
P02746	Complement C1q subcomponent subunit B OS=Homo sapiens OX=9606 GN=C1QB PE=1 SV=3	20	88	21	253	26.7
P0DOY2	Immunoglobulin lambda constant 2 OS=Homo sapiens OX=9606 GN=IGLC2 PE=1 SV=1	15	76	15	106	11.3
P07357	Complement component C8 alpha chain OS=Homo sapiens OX=9606 GN=C8A PE=1 SV=2	39	76	39	584	65.1
P01024	Complement C3 OS=Homo sapiens OX=9606 GN=C3 PE=1 SV=2	43	70	48	1663	187
P17538	Chymotrypsinogen B OS=Homo sapiens OX=9606 GN=CTRB1 PE=2 SV=2	2	68	7	263	27.7
Q6GPI1	Chymotrypsinogen B2 OS=Homo sapiens OX=9606 GN=CTRB2 PE=2 SV=2	2	68	7	263	27.9
P02751	Fibronectin OS=Homo sapiens OX=9606 GN=FN1 PE=1 SV=4	47	67	48	2386	262.5
P00450	Ceruloplasmin OS=Homo sapiens OX=9606 GN=CP PE=1 SV=1	38	56	42	1065	122.1
P36955	Pigment epithelium-derived factor OS=Homo sapiens OX=9606 GN=SERPINF1 PE=1 SV=4	29	54	29	418	46.3
O43866	CD5 antigen-like OS=Homo sapiens OX=9606 GN=CD5L PE=1 SV=1	30	52	30	347	38.1
P02747	Complement C1q subcomponent subunit C OS=Homo sapiens OX=9606 GN=C1QC PE=1 SV=3	16	50	16	245	25.8
P01860	Immunoglobulin heavy constant gamma 3 OS=Homo sapiens OX=9606 GN=IGHG3 PE=1 SV=2	11	45	11	377	41.3
P02745	Complement C1q subcomponent subunit A OS=Homo sapiens OX=9606 GN=C1QA PE=1 SV=2	14	39	15	245	26
P01215	Glycoprotein hormones alpha chain OS=Homo sapiens OX=9606 GN=CGA PE=1 SV=1	2	34	9	116	13.1
P01009	Alpha-1-antitrypsin OS=Homo sapiens OX=9606 GN=SERPINA1 PE=1 SV=3	17	34	18	418	46.7
P0DOX2	Immunoglobulin alpha-2 heavy chain OS=Homo sapiens OX=9606 PE=1 SV=2	13	33	13	455	48.9
P00738	Haptoglobin OS=Homo sapiens OX=9606 GN=HP PE=1 SV=1	15	30	15	406	45.2
A0A0A0MS14	Immunoglobulin heavy variable 1-45 OS=Homo sapiens OX=9606 GN=IGHV1-45 PE=3 SV=1	5	30	5	117	13.5

P01619	Immunoglobulin kappa variable 3-20 OS=Homo sapiens OX=9606 GN=IGKV3-20 PE=1 SV=2	11	30	8	116	12.5
P01701	Immunoglobulin lambda variable 1-51 OS=Homo sapiens OX=9606 GN=IGLV1-51 PE=1 SV=2	9	30	9	117	12.2
P0DOX8	Immunoglobulin lambda-1 light chain OS=Homo sapiens OX=9606 PE=1 SV=1	10	27	10	216	22.8
Q03591	Complement factor H-related protein 1 OS=Homo sapiens OX=9606 GN=CFHR1 PE=1 SV=2	19	27	19	330	37.6
P01225	Follitropin subunit beta OS=Homo sapiens OX=9606 GN=FSHB PE=1 SV=2	3	26	10	129	14.7
P02790	Hemopexin OS=Homo sapiens OX=9606 GN=HPX PE=1 SV=2	11	23	14	462	51.6
P02647	Apolipoprotein A-I OS=Homo sapiens OX=9606 GN=APOA1 PE=1 SV=1	15	21	16	267	30.8
P07360	Complement component C8 gamma chain OS=Homo sapiens OX=9606 GN=C8G PE=1 SV=3	14	21	15	202	22.3
A0A075B6P5	Immunoglobulin kappa variable 2-28 OS=Homo sapiens OX=9606 GN=IGKV2-28 PE=3 SV=1	12	20	12	120	12.9
P02743	Serum amyloid P-component OS=Homo sapiens OX=9606 GN=APCS PE=1 SV=2	12	18	13	223	25.4
P06312	Immunoglobulin kappa variable 4-1 OS=Homo sapiens OX=9606 GN=IGKV4-1 PE=1 SV=1	10	18	10	121	13.4
P01704	Immunoglobulin lambda variable 2-14 OS=Homo sapiens OX=9606 GN=IGLV2-14 PE=1 SV=2	6	16	6	120	12.6
A0A0A0MRZ8	Immunoglobulin kappa variable 3D-11 OS=Homo sapiens OX=9606 GN=IGKV3D-11 PE=3 SV=6	5	14	6	115	12.6
P04433	Immunoglobulin kappa variable 3-11 OS=Homo sapiens OX=9606 GN=IGKV3-11 PE=1 SV=1	5	14	6	115	12.6
P01624	Immunoglobulin kappa variable 3-15 OS=Homo sapiens OX=9606 GN=IGKV3-15 PE=1 SV=2	7	13	7	115	12.5
P02749	Beta-2-glycoprotein 1 OS=Homo sapiens OX=9606 GN=APOH PE=1 SV=3	9	12	9	345	38.3
A0A0B4J1X5	Immunoglobulin heavy variable 3-74 OS=Homo sapiens OX=9606 GN=IGHV3-74 PE=3 SV=1	3	12	3	117	12.8
A0A0B4J1V1	Immunoglobulin heavy variable 3-21 OS=Homo sapiens OX=9606 GN=IGHV3-21 PE=1 SV=1	5	12	4	117	12.8
P23083	Immunoglobulin heavy variable 1-2 OS=Homo sapiens OX=9606 GN=IGHV1-2 PE=1 SV=2	5	12	5	117	13.1
P01700	Immunoglobulin lambda variable 1-47 OS=Homo sapiens OX=9606 GN=IGLV1-47 PE=1 SV=2	6	10	6	117	12.3
Q969X2	Alpha-N-acetylgalactosaminide alpha-2.6-sialyltransferase 6 OS=Homo sapiens OX=9606 GN=ST6GALNAC6 PE=1 SV=1	2	10	2	333	38
P02766	Transthyretin OS=Homo sapiens OX=9606 GN=TTR PE=1 SV=1	6	10	6	147	15.9
P00751	Complement factor B OS=Homo sapiens OX=9606 GN=CFB PE=1 SV=2	7	9	8	764	85.5
P01011	Alpha-1-antichymotrypsin OS=Homo sapiens OX=9606 GN=SERPINA3 PE=1 SV=2	4	7	6	423	47.6
A0A0B4J1V6	Immunoglobulin heavy variable 3-73 OS=Homo sapiens OX=9606 GN=IGHV3-73 PE=3 SV=1	2	5	3	119	12.8
P04217	Alpha-1B-glycoprotein OS=Homo sapiens OX=9606 GN=A1BG PE=1 SV=4	3	5	3	495	54.2

Table S3. Reproducibility of the immunopurification was calculated by measuring the area of peptide ETVRVPGCAHHADSLY representative of the amount of hFSH in each experiment. We report in the table below the intensity of a representative peptide (ETVRVPGCAHHADSLY) in the three IP replicates of spiked samples. The area of the same peptide was extracted from the entire chromatograms. We noticed that the total TICs had a variable intensities, affecting all the peptides in the analysis. For this reason, the peak area was normalised to the TIC intensity.

**Full Scan experiment:**	**XIC Peptide: ETVRVPG-CAHHADSLY**	**m/z = 906.4309**	**z = 2**
**Sample**	**Area**	**Height**	**Norm. Area**
201211_chme_ip_fsh_n1-4_dr_ip_spike_1_10219.raw	14355779	2626075	5.5
201211_chme_ip_fsh_n1-4_dr_ip_spike_2_10220.raw	76709242	11756726	6.5
201211_ChME_IP_FSH_n1-4_DR_IP_spike_3_10221.raw	8494184	1491645	5.7

Table S4. PRM-MS identification of IP_hFSH serum sample. Mass spectrometry data were analysed by Proteome Discoverer v2.4 (Thermo Scientific) against human subset from UniProtKB/Swiss-Prot complete proteome database using the Byonic node search engine (v3.6.0 Protein Metrics. PMI-Suite). Each identified glycopeptides were further validate by interpreting MS/MS spectrum.

***N*-Glycan Site**	**Peptide < ProteinMetrics Confidential >**	**Glycans Composition**	**Glycans Short name**	**Observed m/z**	**z**	**Observed (M+H)**	**Calc. Mass (M+H)**	**Scan Time**	**Note**
N52	L.VQKN[+1913.677]VTSESTCCVAKSY.N	HexNAc(4)Hex(5)NeuAc(1)	A2G2S1	1292.2	3	3874.585	3874.587	19.03	
N78	F.KVEN[+2204.772]HTACHC-STCY.Y	HexNAc(4)Hex(5)NeuAc(2)	A2G2S2	993.625	4	3971.478	3971.477	14.41	
N7	L.TN[+2715.962]ITIAIE-KEECRF.C	HexNAc(5)Hex(6)Fuc(1)NeuAc(2)	FA3G3S2	1480.69	3	4440.07	4439.83	28.17	NO MS/MS
N24	F.CISIN[+1913.677]TTW.C	HexNAc(4)Hex(5)NeuAc(1)	A2G2S1	1454.578	2	2908.149	2908.143	43.39	
	F.CISIN[+1930.692]TTW.C	HexNAc(4)Hex(6)Fuc(1)	FA2G3	975.727	3	2925.166	2925.159	44.06	
	F.CISIN[+2205.793]TTW.C	HexNAc(4)Hex(5)Fuc(2)NeuAc(1)	F2A2G2S1	1600.13	2	3199.253	3200.259	43.46	
	F.CISIN[+2350.830]TTW.C	HexNAc(4)Hex(5)Fuc(1)NeuAc(2)	FA2G2S2	1115.77	3	3345.295	3345.297	49.6	
	F.CISIN[+2424.867]TTW.C	HexNAc(5)Hex(6)Fuc(1)NeuAc(1)	FA3G3S1	1140.455	3	3419.35	3419.333	19.55	NO MS/MS
	F.CISIN[+2643.941]TTW.C	HexNAc(6)Hex(7)NeuAc(1)	A3G4S1	1819.202	2	3637.397	3638.408	47.48	

Table S5. Comparison of identified glycopeptides between all performed experiments: Full scan LC-MS/MS and MS-PRM on IP_hFSH and MS-PRM on IP_hFSH_Glycoenrich.

Peptide Sequence	Glycan Site	*N*-Glycan Composition	*N*-Glycan Short Name	RHS_FSH PRM	IP_hFHS Full Scan	IP_hFHS PRM	IP_hFHS enrich PRM	Note
L.RSKKTMLVQKN*VTSESTCCVAKSY.N	N52	HexNAc(4)Hex(5)NeuAc(2)	A2G2S2	x				
		HexNAc(4)Hex(5)NeuAc(1)	A2G2S1	x				
L.VQKN*VTSESTCCVAKSY.N		HexNAc(4)Hex(5)NeuAc(1)	A2G2S1	x		x	x	
		HexNAc(5)Hex(6)NeuAc(3)	A3G3S3	x				NO MS/MS
		HexNAc(4)Hex(5)NeuAc(2)	A2G2S2	x				
		HexNAc(2)Hex(6)Phospho(1)	Man6_Ph	x				
Y.NRVTVMGGFKVEN*HTACHCSTCY.Y	N78	HexNAc(4)Hex(5)NeuAc(2)	A2G2S2	x				
F.KVEN*HTACHCSTCY.Y		HexNAc(5)Hex(6)NeuAc(2)	A3G3S2	x				
		HexNAc(4)Hex(5)NeuAc(2)	A2G2S2	x		x	x	
		HexNAc(4)Hex(5)NeuAc(1)	A2G2S1	x			x	
L.TN*ITIAIE-KEECRF.C	N7	HexNAc(5)Hex(6)Fuc(1)NeuAc(2)	FA3G3S2	x		x		NO MS/MS
		HexNAc(6)Hex(5)Fuc(1)NeuAc(1)	FA4G2S1	x			x	NO MS/MS
		HexNAc(4)Hex(5)NeuAc(2)	A2G2S2	x				NO MS/MS
		HexNAc(4)Hex(5)Fuc(1)NeuAc(2)	FA2G2S2	x			x	NO MS/MS
F.CISIN*TTW.C	N24	HexNAc(4)Hex(5)Fuc(2)NeuAc(1)	F2A2G2S1	x			x	
		HexNAc(4)Hex(5)NeuAc(2)	A2G2S2	x		x	x	
		HexNAc(5)Hex(6)Fuc(1)NeuAc(2)	FA3G3S2	x			x	
		HexNAc(6)Hex(7)NeuAc(1)	A3G4S1	x		x	x	
		HexNAc(4)Hex(5)Fuc(1)NeuAc(2)	FA2G2S2	x	x	x	x	
		HexNAc(4)Hex(6)Fuc(1)	FA2G2Gal_1	x		x	x	
		HexNAc(4)Hex(5)Fuc(2)NeuAc(2)	F2A2G2S2	x				
		HexNAc(4)Hex(5)Fuc(1)NeuAc(1)	FA2G2S1	x				
		HexNAc(4)Hex(3)	A2G0	x				
		HexNAc(4)Hex(6)Fuc(2)	F2A2G2Gal_1	x				
		HexNAc(4)Hex(5)NeuAc(1)	A2G2S1	x		x	x	
		HexNAc(4)Hex(5)Fuc(1)	FA2G2	x				
		HexNAc(5)Hex(6)Fuc(1)NeuAc(1)	FA3G3S1			x	x	NO MS/MS
		HexNAc(2)Hex(6)Phospho(1)	Man3_Ph	x				

Table S6. Glycopeptides relative quantification in RHS_FSH and IP_hFSH serum sample. The quantifier (table S7) peack area from chromatographic traces post-acquisition extraction are recorded by skyline software. For each *N*-glycan site is reported the relative percentage abundance.

RT (min)	Peptides Sequence	*N*-Glycan Site	Gycan	RHS_FSH (Peack Area Quantifier)	IP_hFSH (Peack Area Quantifier)	RHS_FSH Site-Specific Distribution (%)	IP_hFSH Site-Specific Distribution (%)
23.24	RSKKTMLVQKN[+1914]VTSESTCCVAKSY	N52	A2G2S1	2.00E+06	N.D.	20.64	0.00
24.16	RSKKTMLVQKN[+2205]VTSESTCCVAKSY		A2G2S2	1.97E+06	N.D.	20.33	0.00
20.96	VQKN[+1458.4]VTSESTCCVAKSY		MAN6_Ph	2.11E+05	N.D.	2.18	0.00
24.26	VQKN[+2205]VTSESTCCVAKSY		A2G2S2	1.83E+06	N.D.	18.88	0.00
20.84	VQKN[+1914]VTSESTCCVAKSY		A2G2S1	3.68E+06	7.48E+05	37.97	100
27.12	NRVTVMGGFKVEN[+2205]HTACHCSTCY	N78	A2G2S2	1.77E+06	N.D.	1.47	0.00
14.43	KVEN[+1914]HTACHCSTCY		A2G2S1	9.92E+06	N.D.	8.25	0.00
15.85	KVEN[+2205]HTACHCSTCY		A2G2S2	1.06E+08	2.70E+05	88.14	100
15.78	KVEN[+2569.9]HTACHCSTCY		A3G3S2	2.57E+06	N.D.	2.14	0.00
47.54	C[+57]ISIN[+2205]TTW	N24	A2G2S2	4.75E+05	8.32E+05	1.01	45.34
47.5	C[+57]ISIN[+2716]TTW		FA3G3S2	9.56E+05	N.D.	1.50	0.00
47.51	C[+57]ISIN[+2642.9]TTW		A3G4S1	7.18E+05	1.04E+05	1.13	7.25
49.61	C[+57]ISIN[+2351]TTW		FA2G2S2	5.60E+07	4.29E+05	87.99	29.90
45.09	C[+57]ISIN[+1930.7]TTW		FA2G2_Gal1	4.77E+04	7.93E+04	0.07	5.53
47.51	C[+57]ISIN[+2496.9]TTW		F2A2G2S2	7.97E+05	N.D.	1.25	0.00
44.08	C[+57]ISIN[+1298.5]TTW		A2G0	2.75E+05	N.D.	0.43	0.00
44.72	C[+57]ISIN[+2076.8]TTW		F2A2G3	4.28E+05	N.D.	0.67	0.00
47.52	C[+57]ISIN[+1914]TTW		A2G2S1	2.20E+05	1.72E+05	0.35	11.99
36.93	C[+57]ISIN[+1768.6]TTW		FA2G2	1.26E+06	N.D.	1.98	0.00
47.49	C[+57]ISIN[+1458.4]TTW		MAN6_Ph	1.47E+05	N.D.	0.23	0.00
44.7	C[+57]ISIN[+2060]TTW		FA2G2S1	1.68E+06	N.D.	2.64	0.00

N.D. = Not Detected.

Table S7. Glycopeptides transitions (Precursor Mz → Product Mz) were selected post-data acquisition by PRM-MS analysis. Glycopeptides were identified by coelution of at least 3 transitions and the highest one (*) was selected as quantifier.

N-glycan site	Peptide Modified Sequence	Glycan	Precursor Mz	Precursor Charge	Product Mz	Product Charge	Fragment Ion Description
N52	RSKKTMLVQKN[+1914]VTSEST CCVAKSY	A2G2S1	944.6869	5	292.08	1	NeuAc
					366.14 *	1	Hex+HexNac
					528.19	1	HexNac+2Hex
					1505.27	2	Pep+HexNac
	RSKKTMLVQKN[+2205]VTSEST CCVAKSY	A2G2S2	1253.357	4	292.08	1	NeuAc
					366.14 *	1	Hex+HexNac
					528.19	1	HexNac+2Hex
					1505.27	2	Pep+HexNac
	VQKN[+1458.4]VTSESTCCVAKS Y	MAN6_P	1140.456	3	204.09	1	HexNac
					243.03	1	Hex_P
					366.14 *	1	Hex+HexNac
					405.08	1	2Hex_P
	VQKN[+2205]VTSESTCCVAKSY	A2G2S2	1389.308	3	204.09	1	HexNac
					292.08	1	NeuAc
					366.14 *	1	Hex+HexNac
					1391.58	2	y12
	VQKN[+1914]VTSESTCCVAKSY	A2G2S1	1292.308	3	204.09	1	HexNac
					274.09 *	1	NeuAc-18
					292.08	1	NeuAc
					366.14	1	Hex+HexNac
N78	NRVTVMGGFKVEN[+2205]HTA CHCSTCY	A2G2S2	1234.052	4	292.08	1	NeuAc
					366.14 *	1	Hex+HexNac
					512.2	1	Fuc+Hex+HexNac
					1465.66	2	Pep+HexNac
	KVEN[+1914]HTACHCSTCY	A2G2S1	920.9315	4	204.09	1	HexNac
					274.09 *	1	NeuAc-18
					366.14	1	Hex+HexNac
					985.4	2	Pep+HexNac
	KVEN[+2205]HTACHCSTCY	A2G2S2	993.6815	4	274.09 *	1	NeuAc-18
					292.08	1	NeuAc
					366.14	1	Hex+HexNac
					985.4	2	Pep+HexNac
	KVEN[+2569.9]HTACHCSTCY	A3G3S2	1084.908	4	366.14 *	1	Hex+HexNac
					528.19	1	HexNac+2Hex
					985.4	2	Pep+HexNac
N24	CISIN[+2205.8]TTW	F2A2G2S1	1067.424	3	274.09 *	1	NeuAc-18
					366.14	1	Hex+HexNac
					671.31	2	Pep+HexNac+Fuc
					1067.42	3	precursor
	CISIN[+2716]TTW	FA3G3S2	1855.717	2	274.09	1	NeuAc-18
					366.14 *	1	Hex+HexNac
					671.31	2	Pep+HexNac+Fuc
	CISIN[+2642.9]TTW	A3G4S1	1819.207	2	274.09 *	1	NeuAc-18
					292.08	1	NeuAc
					366.14	1	Hex+HexNac
	CISIN[+2351]TTW	FA2G2S2	1115.827	3	274.09 *	1	NeuAc-18
					366.14	1	Hex+HexNac
					671.31	2	Pep+HexNac+Fuc
	CISIN[+1930.7]TTW	FA2G2_Gal 1	975.7243	3	204.09	1	HexNac
					274.12 *	1	b2

					366.14	1	Hex+HexNac
					512.2	1	Fuc+Hex+HexNac
	CISIN[+2496.9]TTW	F2A2G2S2	1746.181	2	274.09 *	1	NeuAc-18
					292.08	1	NeuAc
					366.14	1	Hex+HexNac
	CISIN[+1298.5]TTW	A2G0	1146.975	2	204.09	1	HexNac
					274.12 *	1	b2
					366.14	1	Hex+HexNac
	CISIN[+2076.8]TTW	F2A2G3	1536.112	2	274.12	1	b2
					361.15	1	b3
					366.14 *	1	Hex+HexNac
	CISIN[+1914]TTW	A2G2S1	1454.737	2	204.09	1	HexNac
					292.08	1	NeuAc
					366.14 *	1	Hex+HexNac
	CISIN[+1768.6]TTW	FA2G2	1382.057	2	366.14 *	1	Hex+HexNac
					512.2	1	Fuc+Hex+HexNac
					671.31	2	Pep+HexNac+Fuc
					1382.05	2	precursor
	CISIN[+1458.4]TTW	MAN6_P	1226.958	2	243.03	1	Hex_P
					366.14 *	1	Hex+HexNac
					405.08	1	2Hex_P
					1226.96	2	precursor
	CISIN[+2060]TTW	FA2G2S1	1527.737	2	274.09 *	1	NeuAc-18
					292.08	1	NeuAc
					366.14	1	Hex+HexNac
					671.31	2	Pep+HexNac+Fuc
Unmodified peptide	ETVRVPGCAHHADSLY	-	906.431	2	970.48	1	b9
					1107.54*	1	b10
					715.83	2	b13

Glycopeptide's library

Standard recombinant FSH glycopeptides identified (Table S1) are a key step in our workflow as they constitute a library of retention time (RT), MS (precursor ion) and MS2 (most intense fragment ions) of target glycopeptides included into the target list for the development of PRM-MS method to identify serum hFSH glycopeptides. Each glycopeptide was validated before importing in MS-PRM method by MS2 spectra interpretation using Byonic software (www.proteinmetrics.com). Minority glycoforms were manually validated by inspecting the raw files (data are not showed). Here we showed the detail of the 15 most representative glycopeptides (Table S8) and for each of them we reported: glycopeptides information table, precursor ion XIC and relative MS2 spectrum and MS2 spectrum interpretation by Byonic software.

Table S8. Most intense glycopeptides identified in standard recombinant FSH selected for glycopeptides library.

Protein Name	#	Scan Time	Glycopepeptide	Glycans	Glycan short name	N glycan site	Score	Observed m/z	z	Observed (M+H)	Calc. mass (M+H)
>sp|P01215| GLHA_HUMAN Glycoprotein hormones alpha chain	1	14.43	F.KVEN[+1913.677]HTACHCSTCY.Y	HexNAc(4)Hex(5)NeuAc(1)	A2G2S1	78	635	920.848	4	3680.371	3680.381
	2	15.75	F.KVEN[+2569.905]HTACHCSTCY.Y	HexNAc(5)Hex(6)NeuAc(2)	A3G3S2	78	375	1084.906	4	4336.604	4336.609
	3	15.85	F.KVEN[+2204.772]HTACHCSTCY.Y	HexNAc(4)Hex(5)NeuAc(2)	A2G2S2	78	648	993.624	4	3971.473	3971.477
	4	20.84	L.VQKN[+1913.677]VTSESTCCVAKSY.N	HexNAc(4)Hex(5)NeuAc(1)	A2G2S1	52	496	1292.200	3	3874.586	3874.587
	5	20.95	L.VQKN[+1458.442]VTSESTCCVAKSY.N	HexNAc(2)Hex(6)Phospho(1)	Man6-Ph	52	473	1140.455	3	3419.350	3419.352
	6	23.19	L.RSKKTMLVQKN[+1913.677]VTSESTCCVAKSY.N	HexNAc(4)Hex(5)NeuAc(1)	A2G2S1	52	425	944.621	5	4719.077	4719.083
	7	24.52	L.RSKKTMLVQKN[+2204.772]VTSESTCCVAKSY.N	HexNAc(4)Hex(5)NeuAc(2)	A2G2S2	52	154	1253.298	4	5010.171	5010.178
	8	27.08	Y.NRVTVMGGFKVEN[+2204.772]HTACHCSTCY.Y	HexNAc(4)Hex(5)NeuAc(2)	A2G2S2	78	271	1233.996	4	4932.962	4932.957
>sp|P01225| FSHB_HUMAN Follitropin subunit beta	9	36.97	F.CISIN[+1768.640]TTW.C	HexNAc(4)Hex(5)Fuc(1)	FA2G2	24	332	921.707	3	2763.106	2763.106
	10	44.46	F.CISIN[+2205.793]TTW.C	HexNAc(4)Hex(5)Fuc(2)NeuAc(1)	F2A2G2S1	24	153	1067.424	3	3200.256	3200.259
	11	44.72	F.CISIN[+2076.750]TTW.C	HexNAc(4)Hex(6)Fuc(2)	F2A2G3	24	220	1536.109	2	3071.211	3071.217
	12	44.75	F.CISIN[+2059.735]TTW.C	HexNAc(4)Hex(5)Fuc(1)NeuAc(1)	FA2G2S1	24	311	1527.603	2	3054.198	3054.201
	13	49.34	F.CISIN[+2496.888]TTW.C	HexNAc(4)Hex(5)Fuc(2)NeuAc(2)	F2A2G2S2	24	155	1746.173	2	3491.339	3491.355
	14	49.63	F.CISIN[+2350.830]TTW.C	HexNAc(4)Hex(5)Fuc(1)NeuAc(2)	FA2G2S2	24	481	1115.771	3	3345.298	3345.297
	15	50.06	F.CISIN[+2715.963]TTW.C	HexNAc(5)Hex(6)Fuc(1)NeuAc(2)	FA3G3S2	24	90	1855.721	2	3710.435	3710.429

Glycopeptide #1:

Table S9. Glycopeptide #1 information table. The highest fragment (*) was selected as quantifier.

GLHA_HUMAN Glycoprotein hormones alpha chain							
**RT**	**Glycopepeptide**	***N*-glycan site**	**Glycan**	**Precursor ion**	**z**	**Most intense Fragment ion**	**z**
23.19	L.RSKKTMLVQKN[+1913.677]VTSESTCCVAKSY.N	α Asn 52	A2G2S1	944.622	5	292.08	1
						366.14*	1
						528.19	1
						1505.27	2

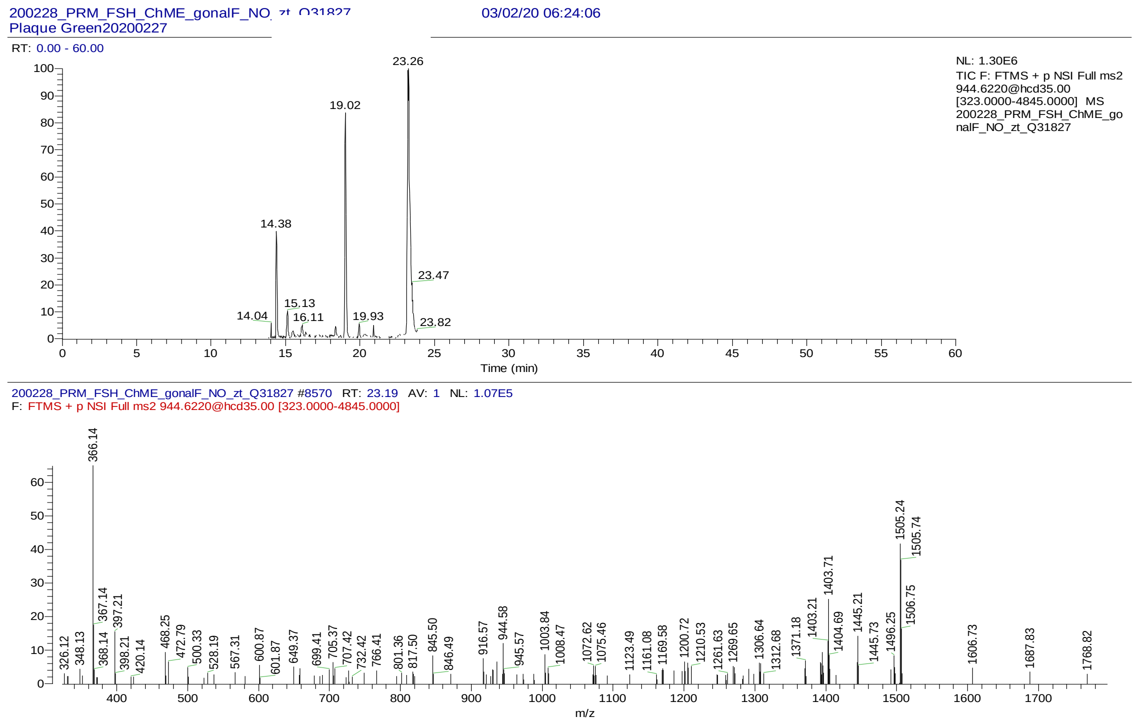

Figure S4. Glycopeptide #1 precursor ion XIC and relative MS2 spectrum.

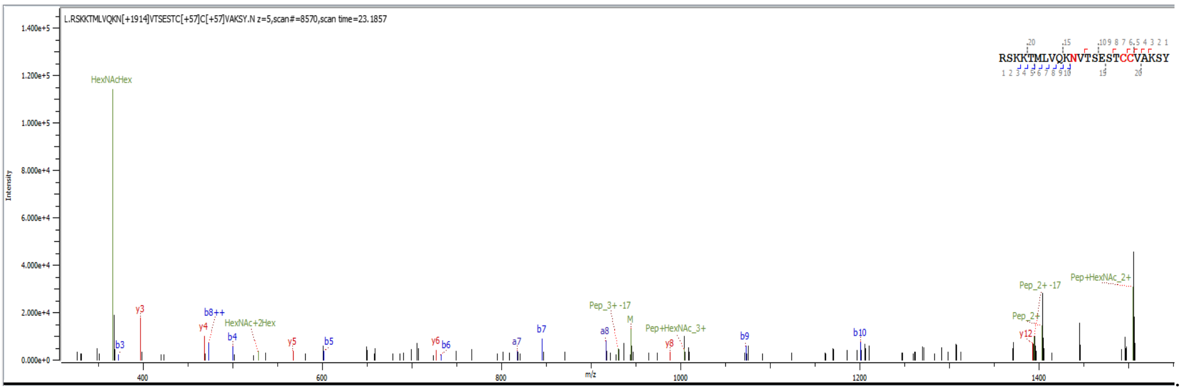

Figure S5. Glycopeptide #1 MS2 spectrum interpretation by Byonic software.

Glycopeptide #2

Table S10. Glycopeptide #2 information table. The highest fragment (*) was selected as quantifier.

GLHA_HUMAN Glycoprotein hormones alpha chain							
RT	Glycopeptide	*N*-glycan site	Glycan	Precursor ion	z	Most intense Fragment ion	z
24.52	L.RSKKTMLVQKN[+2204.772]VTSESTCCVAKSY.N	α Asn 52	A2G2S2	1253.298	4	292.08	1
						366.14*	1
						528.19	1
						1505.27	2

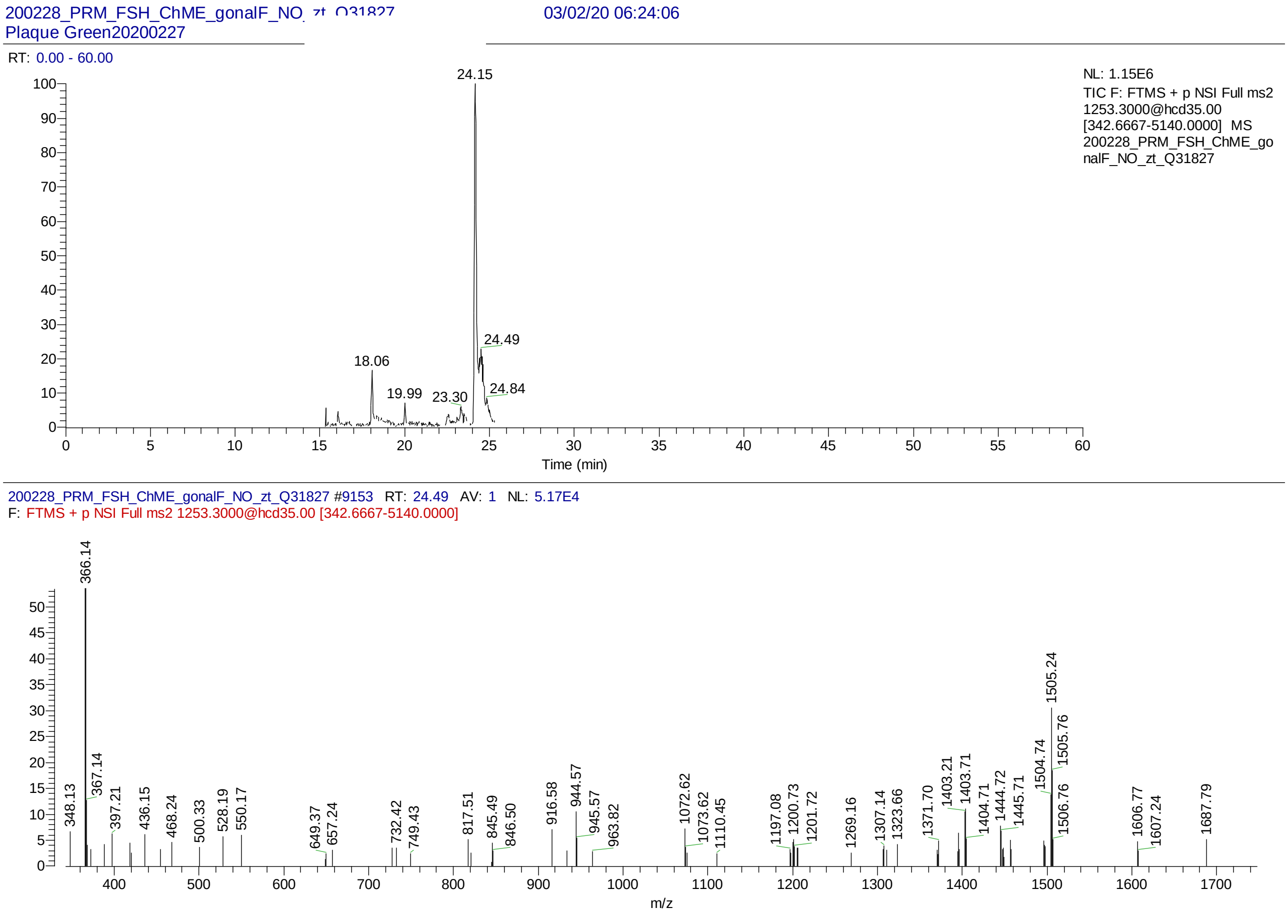

Figure S6. Glycopeptide #2 precursor ion XIC and relative MS2 spectrum.

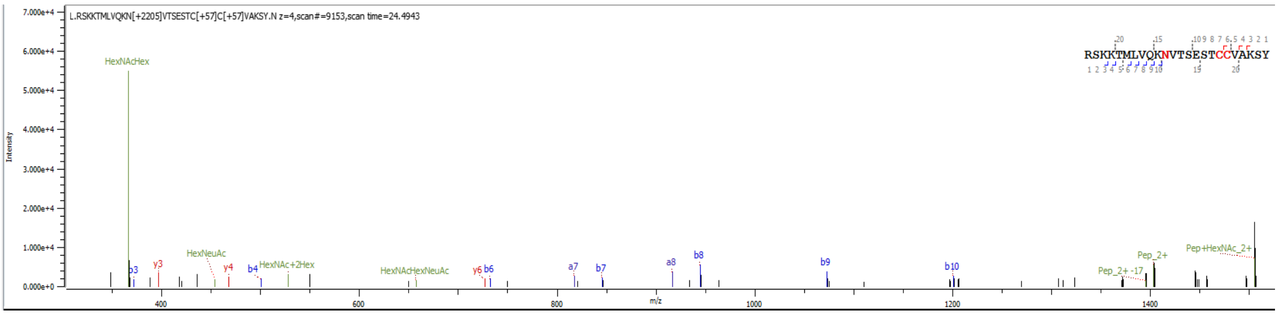

Figure S7. Glycopeptide #2 MS2 spectrum interpretation by Byonic software.

Glycopeptide #3

Table S11. Glycopeptide #3 information table. The highest fragment (*) was selected as quantifier.

GLHA_HUMAN Glycoprotein hormones alpha chain							
**RT**	**Glycopeptide**	***N*-glycan site**	**Glycan**	**Precursor ion**	**z**	**Most intense Fragment ion**	**z**
20.95	L.VQKN[+1458.442]VTSESTCCVAKSY.N	α Asn 52	Man6_Ph	1140.45	3	204.09	1
						243.03*	1
						366.14	1
						405.08	1

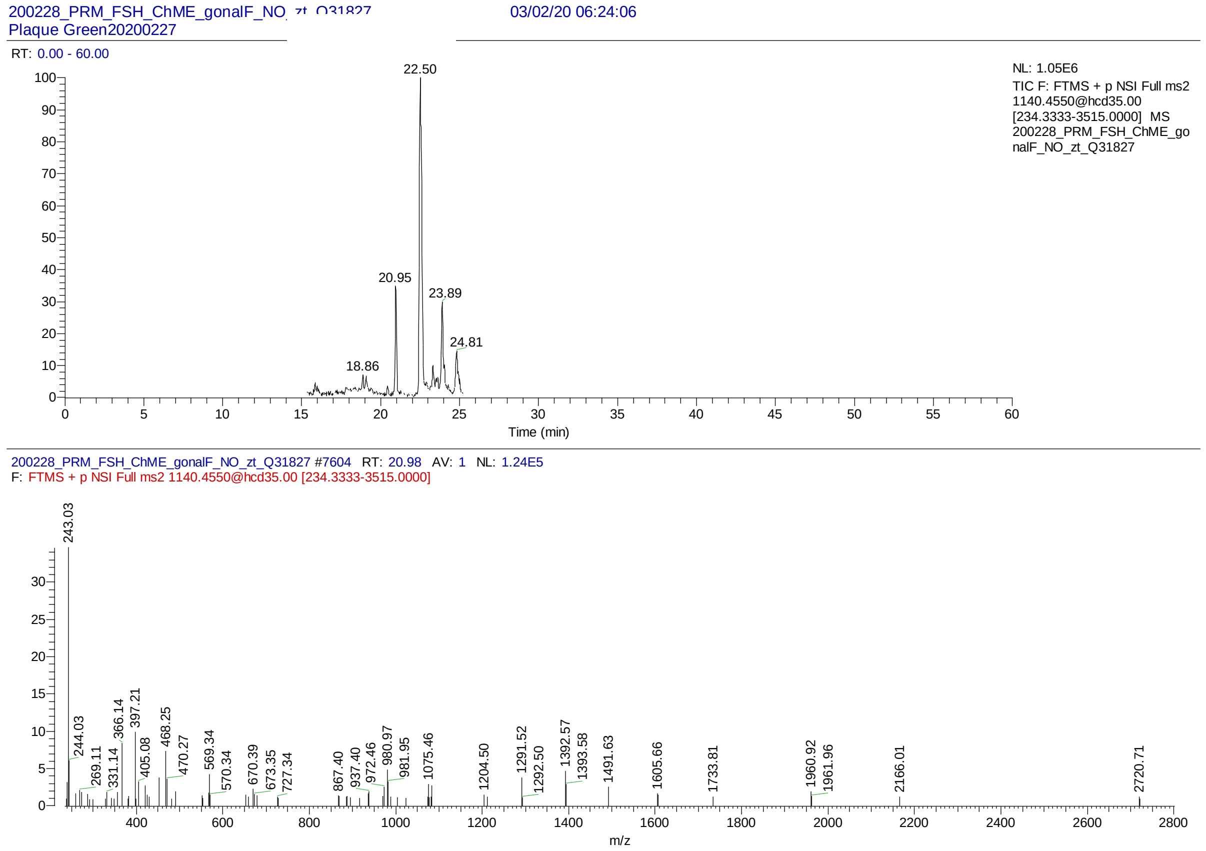

Figure S8. Glycopeptide #3 precursor ion XIC and relative MS2 spectrum.

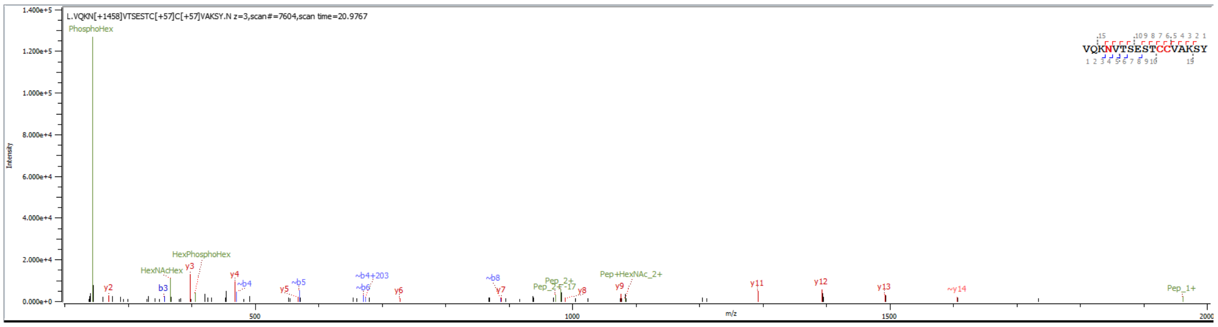

Figure S9. Glycopeptide #3 MS2 spectrum interpretation by Byonic software.

Glycopeptide #4

Table S12. Glycopeptide #4 information table. The highest fragment (*) was selected as quantifier.

GLHA_HUMAN Glycoprotein hormones alpha chain							
**RT**	**Glycopeptide**	***N*-glycan site**	**Glycan**	**Precursor ion**	**z**	**Most intense Fragment ion**	**z**
24.25	L.VQKN[+2204.772]VTSESTCCVAKSY.N	α Asn 52	A2G2S2	1389.229	3	204.09	1
						292.08	1
						366.14*	1
						1391.58	2

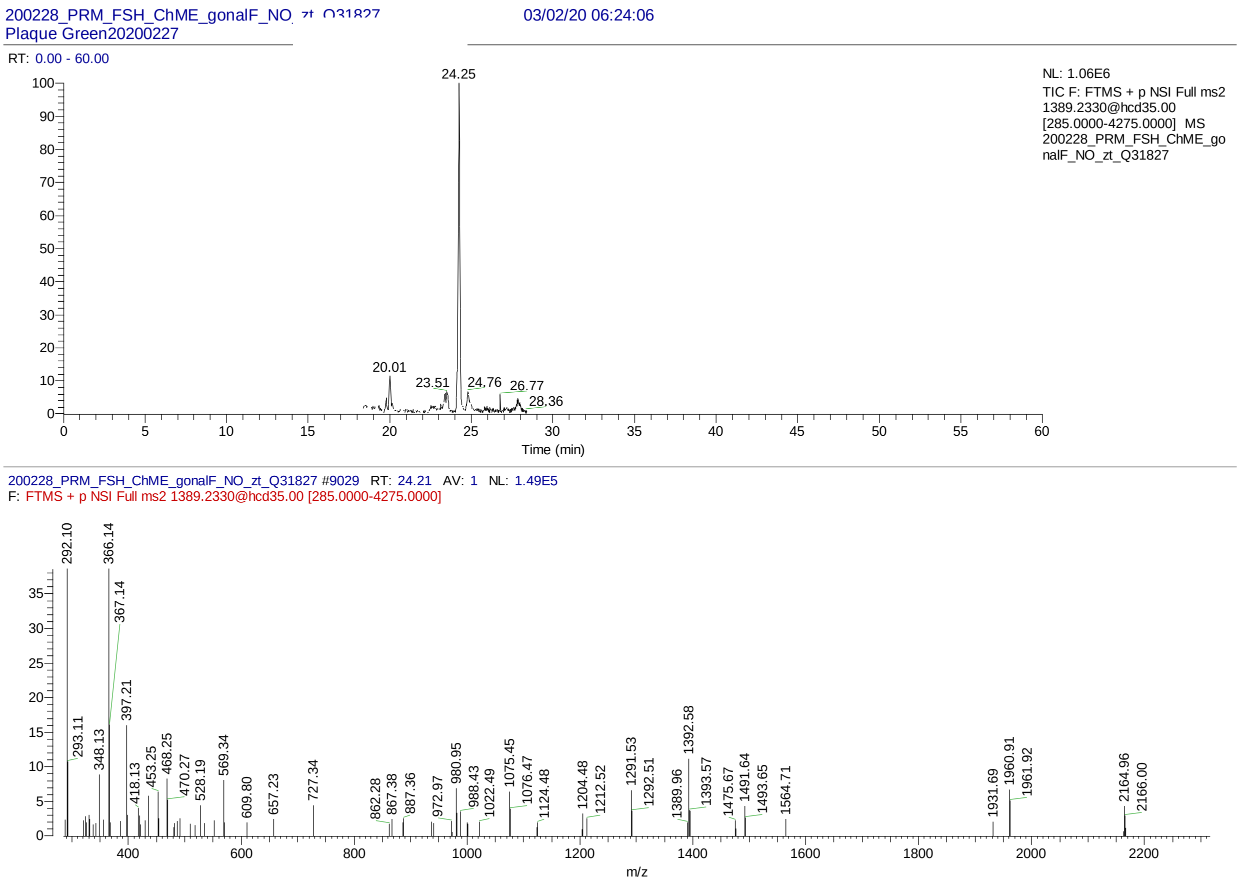

Figure S10. Glycopeptide #4 precursor ion XIC and relative MS2 spectrum.

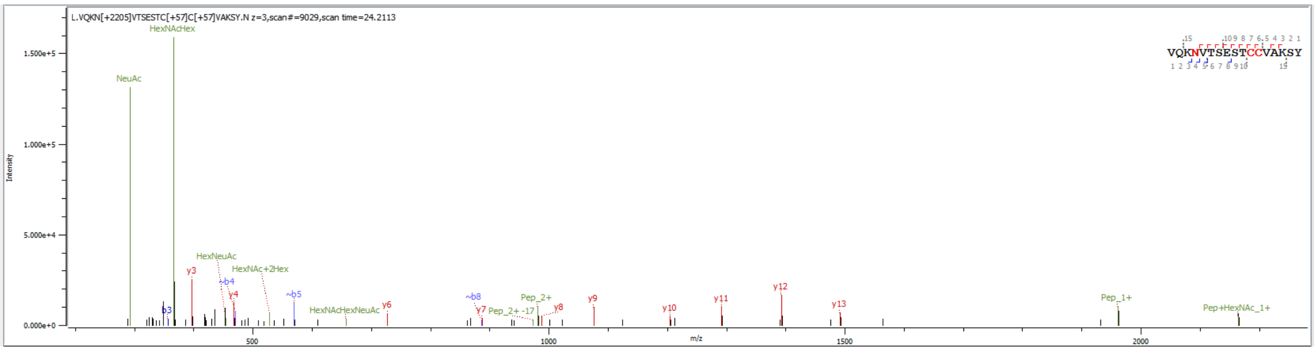

Figure S11. Glycopeptide #4 MS2 spectrum interpretation by Byonic software.

Glycopeptide #5

Table S13. Glycopeptide #5 information table. The highest fragment (*) was selected as quantifier.

GLHA_HUMAN Glycoprotein hormones alpha chain							
**RT**	**Glycopeptide**	***N*-glycan site**	**Glycan**	**Precursor ion**	**z**	**Most intense Fragment ion**	**z**
20.84	L.VQKN[+1913.677]VTSESTCCVAKSY.N	α Asn 52	A2G2S1	1292.200	3	204.09	1
						274.09*	1
						292.08	1
						366.14	1

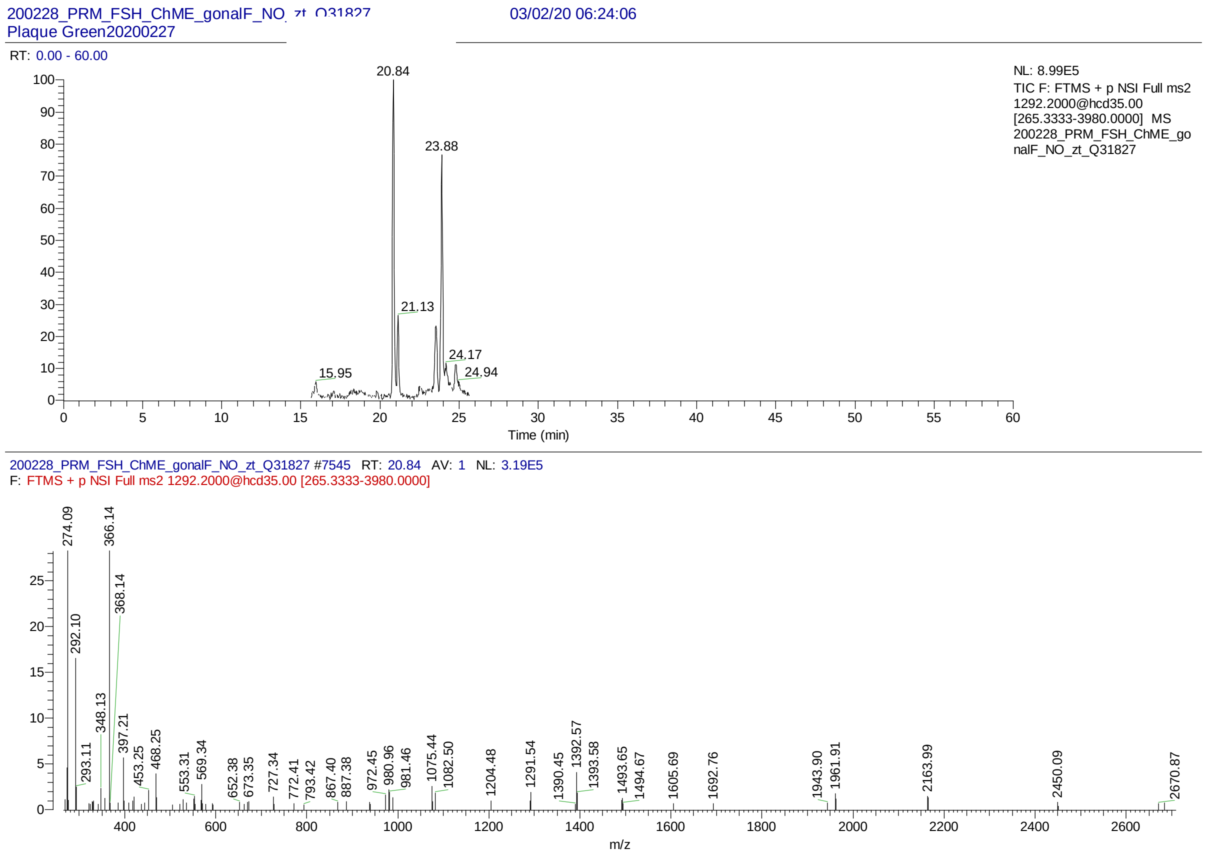

Figure S12. Glycopeptide #5 precursor ion XIC and relative MS2 spectrum.

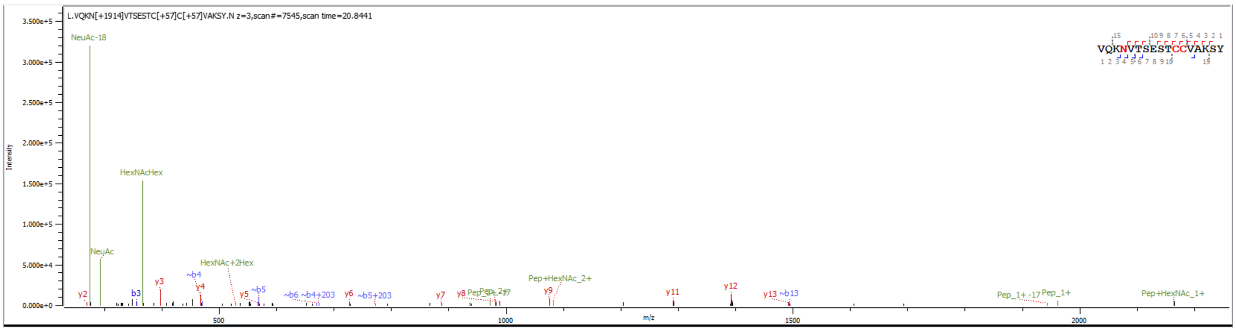

Figure S13. Glycopeptide #5 MS2 spectrum interpretation by Byonic software.

Glycopeptide #6

Table S14. Glycopeptide #6 information table. The highest fragment (*) was selected as quantifier.

GLHA_HUMAN Glycoprotein hormones alpha chain							
**RT**	**Glycopeptide**	***N*-glycan site**	**Glycan**	**Precursor ion**	**z**	**Most intense Fragment ion**	**z**
14.42	F.KVEN[+1913.677]HTACHCSTCY.Y	α Asn 78	A2G2S1	920.848	4	204.09	1
						274.09*	1
						366.14	1
						985.4	2

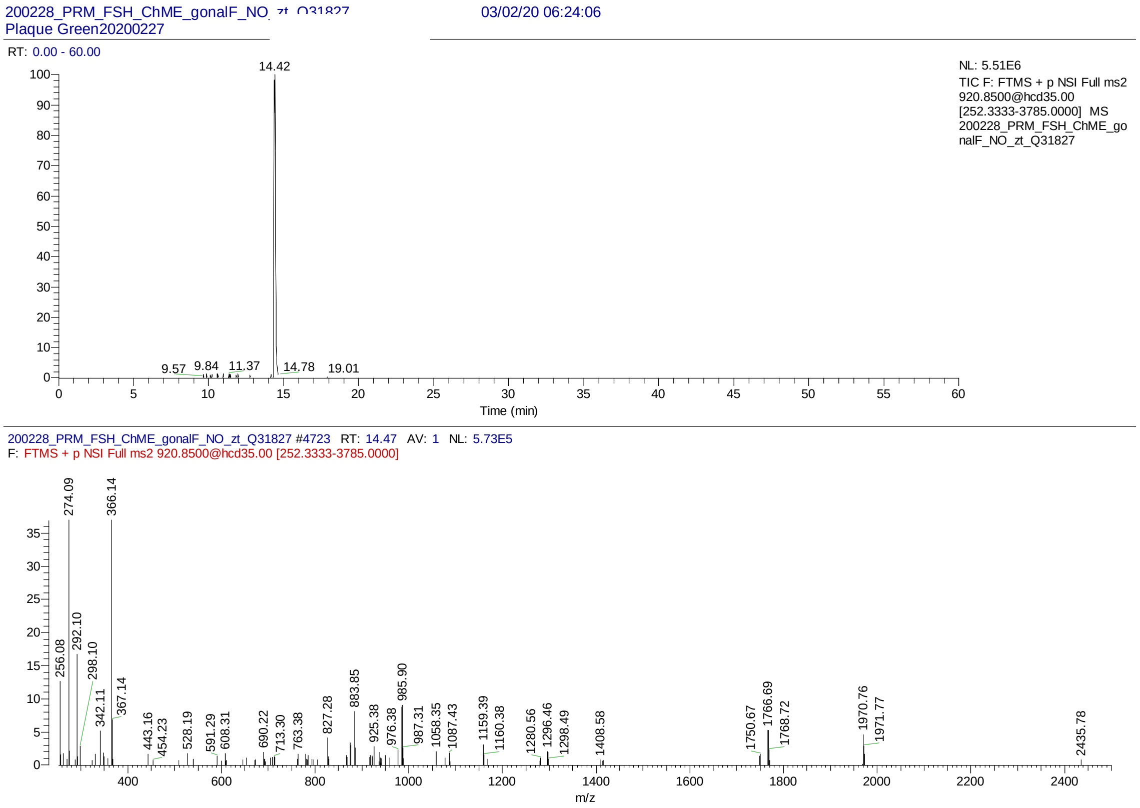

Figure S14. Glycopeptide #6 precursor ion XIC and relative MS2 spectrum.

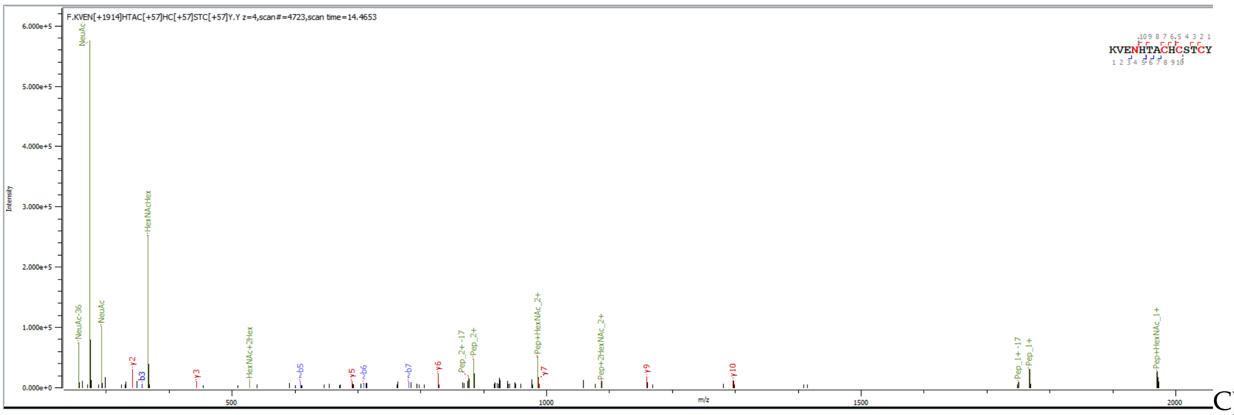

Figure S15. Glycopeptide #6 MS2 spectrum interpretation by Byonic software.

Glycopeptide #7

Table S15. Glycopeptide #7 information table. The highest fragment (*) was selected as quantifier.

GLHA_HUMAN Glycoprotein hormones alpha chain							
**RT**	**Glycopeptide**	**N-glycan site**	**Glycan**	**Precursor ion**	**z**	**Most intense Fragment ion**	**z**
15.85	F.KVEN[+2204.772]HTACHCSTCY.Y	α Asn 78	A2G2S2	993.625	4	274.09*	1
						292.08	1
						366.14	1
						985.4	2

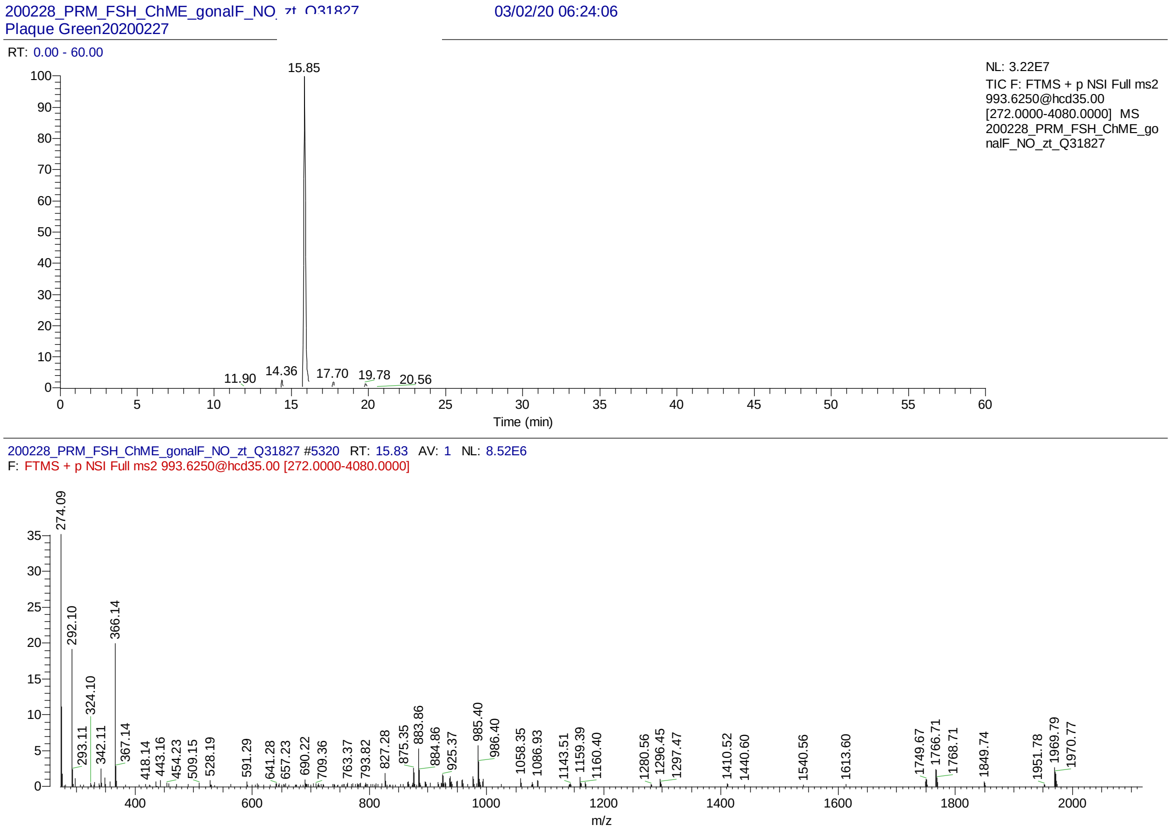

Figure S16. Glycopeptide #7 precursor ion XIC and relative MS2 spectrum.

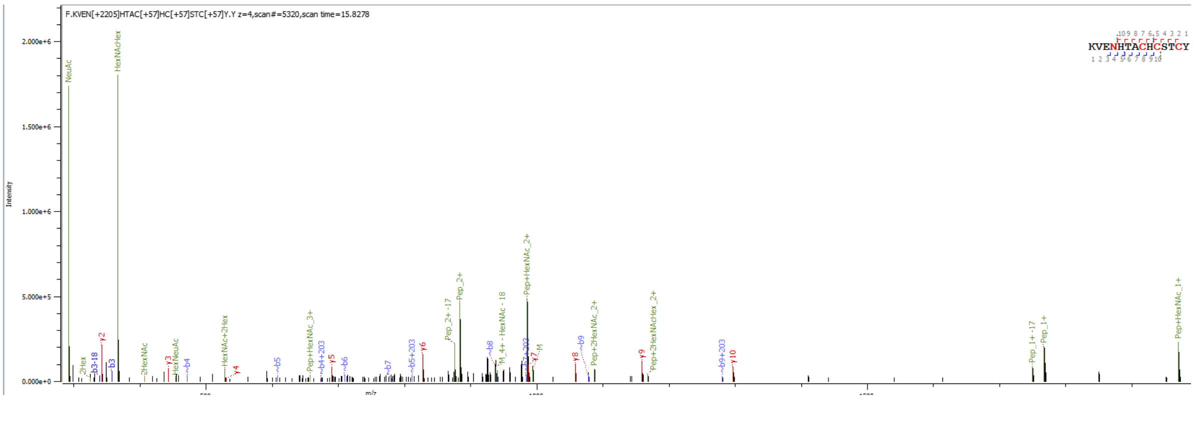

Figure S17. Glycopeptide #7 MS2 spectrum interpretation by Byonic software.

Glycopeptide #8

Table S16. Glycopeptide #8 information table. The highest fragment (*) was selected as quantifier.

GLHA_HUMAN Glycoprotein hormones alpha chain							
**RT**	**Glycopeptide**	***N*-glycan site**	**Glycan**	**Precursor ion**	**z**	**Most intense Fragment ion**	**z**
15.75	F.KVEN[+2569.905]HTACHCSTCY.Y	α Asn 78	A3G3S2	1084.906	4	366.14*	1
						528.19	1
						985.4	2

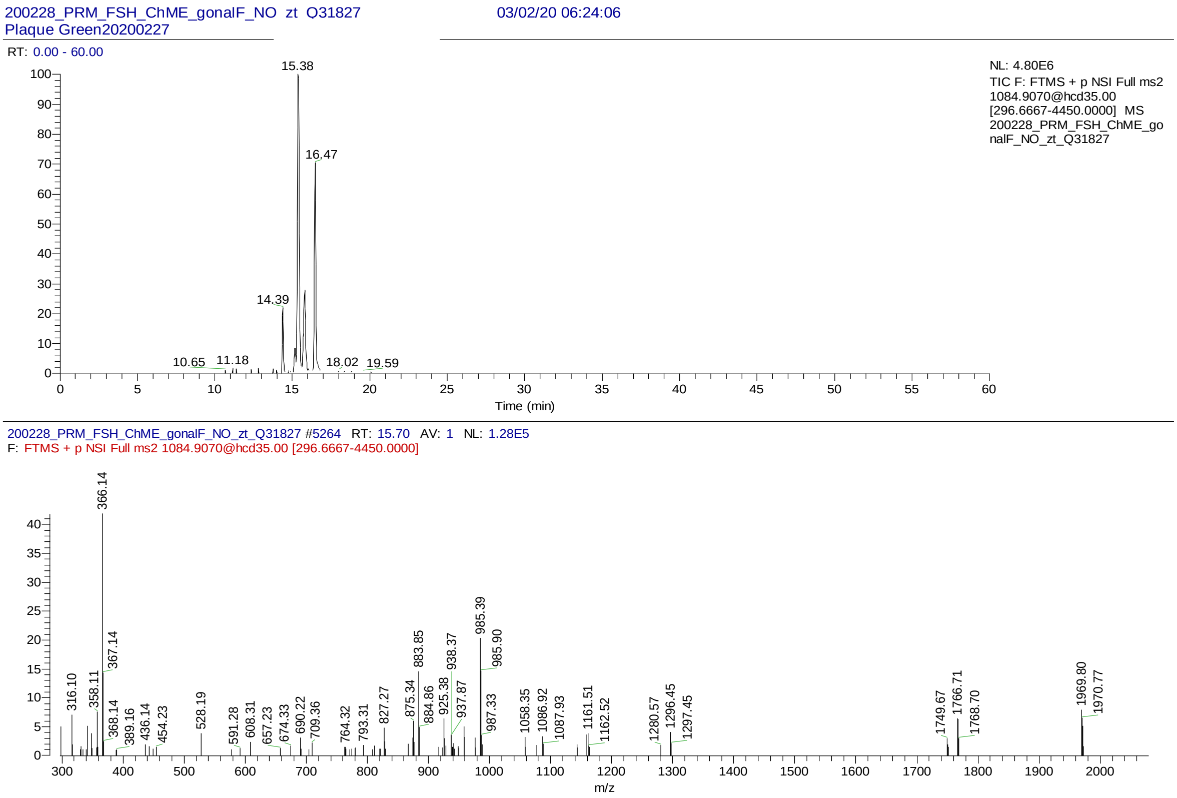

Figure S18. Glycopeptide #8 precursor ion XIC and relative MS2 spectrum.

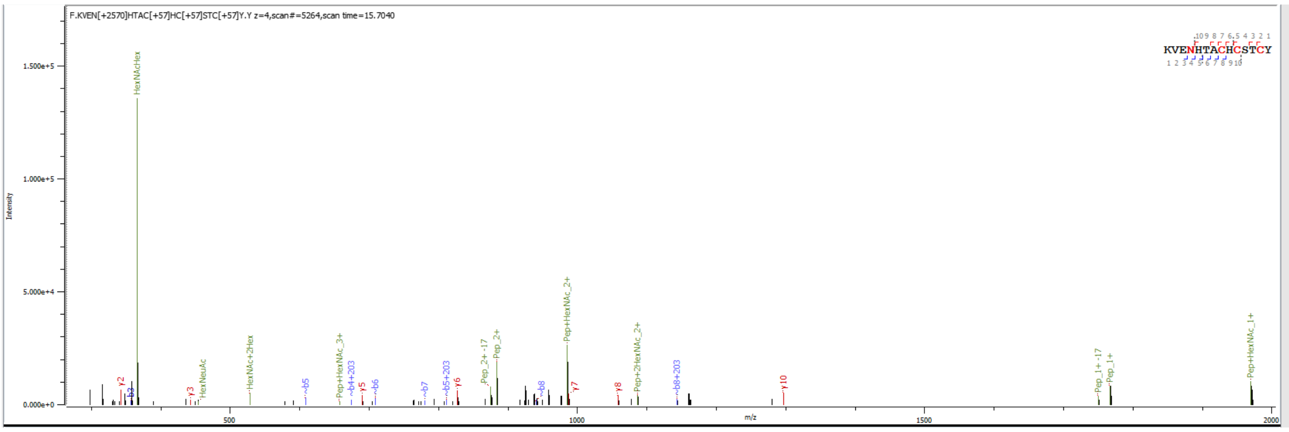

Figure S19. Glycopeptide #8 MS2 spectrum interpretation by Byonic software.

Glycopeptide # 9

Table 17. Glycopeptide #9 information table. The highest fragment (*) was selected as quantifier.

FSHB_HUMAN Follitropin subunit beta							
**RT**	**Glycopeptide**	***N*-glycan site**	**Glycan**	**Precursor ion**	**z**	**Most intense Fragment ion**	**z**
44.46	F.CISIN[+2205.793]TTW.C	b Asn 24	F2A2G2S1	1067.424	3	274.09*	1
						366.14	1
						671.31	2
						1067.42	3

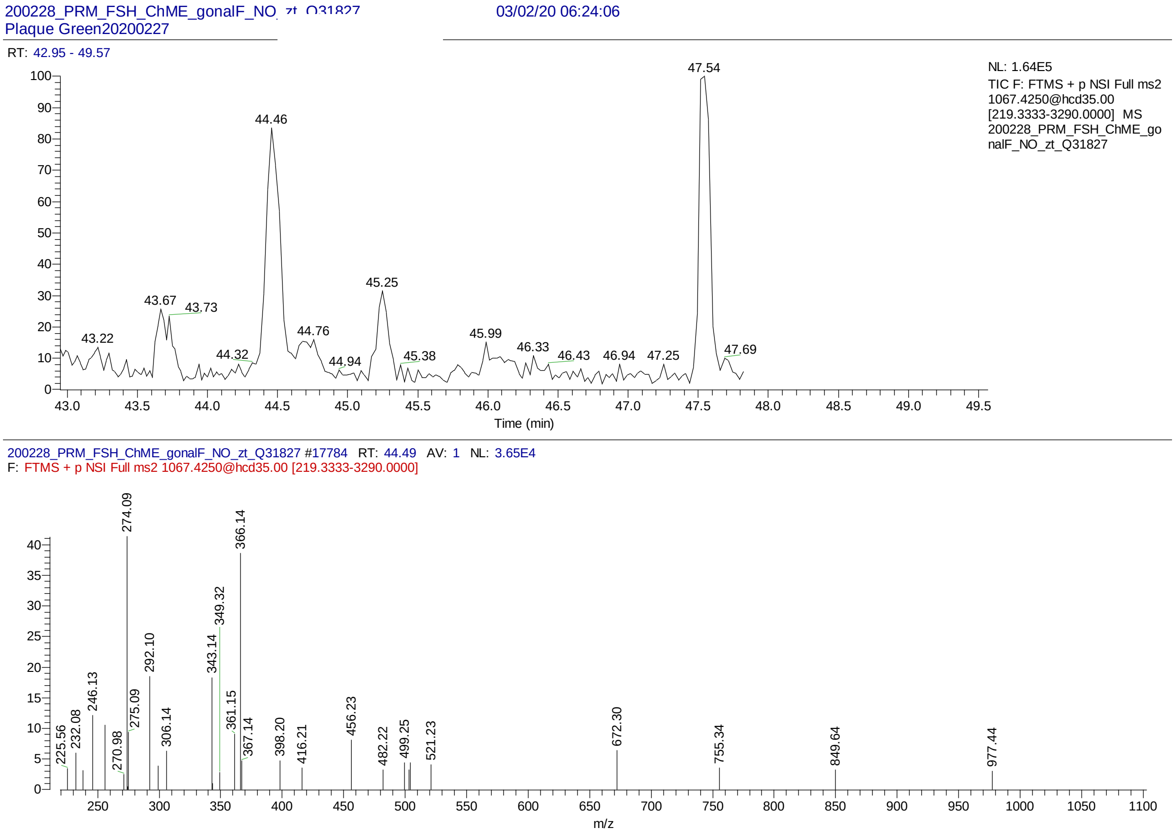

Figure S20. Glycopeptide #9 precursor ion XIC and relative MS2 spectrum.

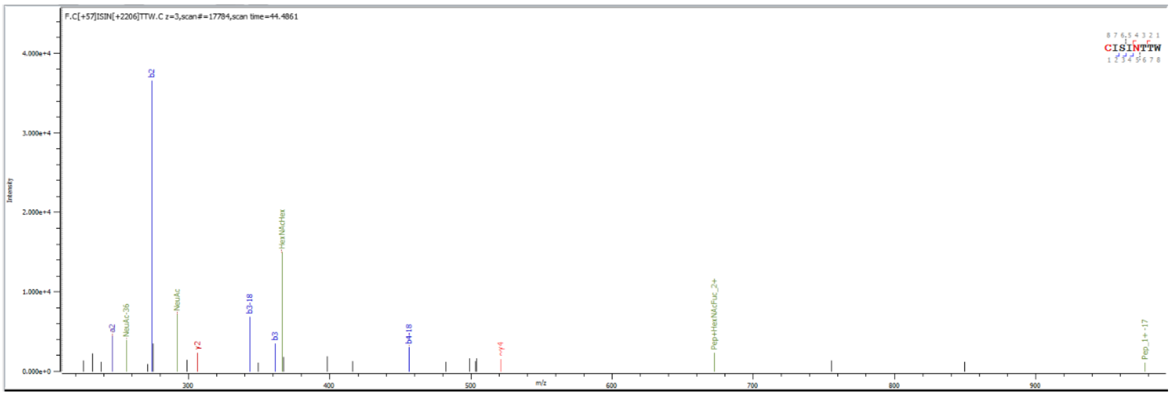

Figure S21. Glycopeptide #9 MS2 spectrum interpretation by Byonic software.

Glycopeptide #10

Table S18. Glycopeptide #10 information table. The highest fragment (*) was selected as quantifier.

FSHB_HUMAN Follitropin subunit beta							
**RT**	**Glycopeptide**	***N*-glycan site**	**Glycan**	**Precursor ion**	**z**	**Most intense Fragment ion**	**z**
50.06	F.CISIN[+2715.963]TTW.C	b Asn 24	FA3G3S2	1855.721	2	274.09	1
						366.14*	1
						671.31	2
						274.09	1

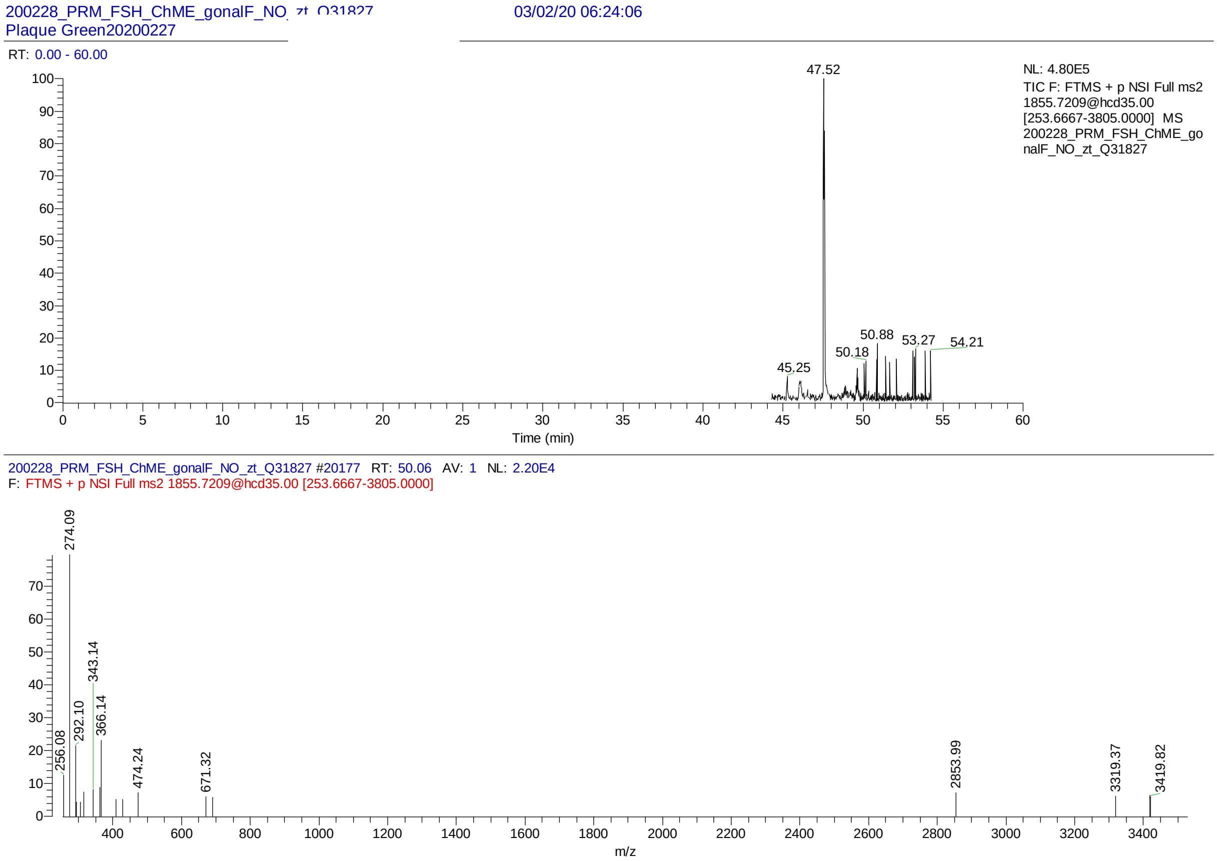

Figure S22. Glycopeptide #10 precursor ion XIC and relative MS2 spectrum.

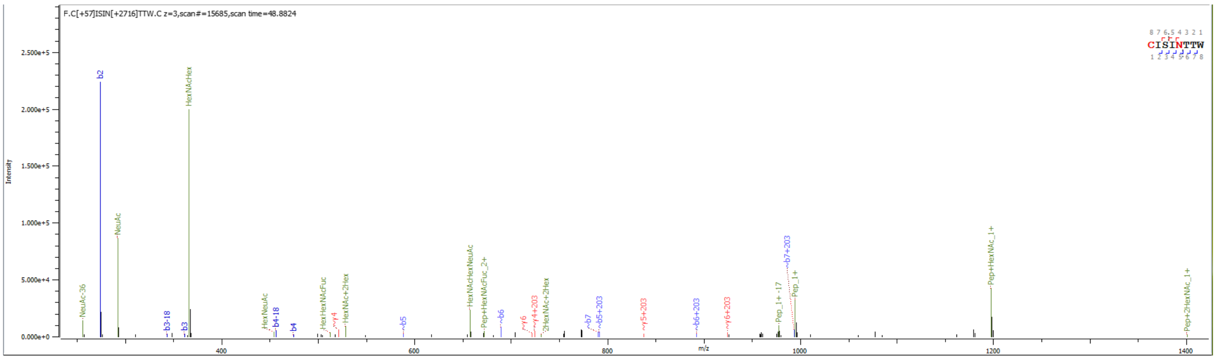

Figure S23. Glycopeptide #10 MS2 spectrum interpretation by Byonic software.

Glycopeptide #11

Table S19. Glycopeptide #11 information table. The highest fragment (*) was selected as quantifier.

FSHB_HUMAN Follitropin subunit beta							
**RT**	**Glycopeptide**	***N*-glycan site**	**Glycan**	**Precursor ion**	**z**	**Most intense Fragment ion**	**z**
49.63	F.CISIN[+2350.830]TTW.C	b Asn 24	FA2G2S2	1115.771	3	274.09*	1
						366.14	1
						671.31	2
						274.09*	1

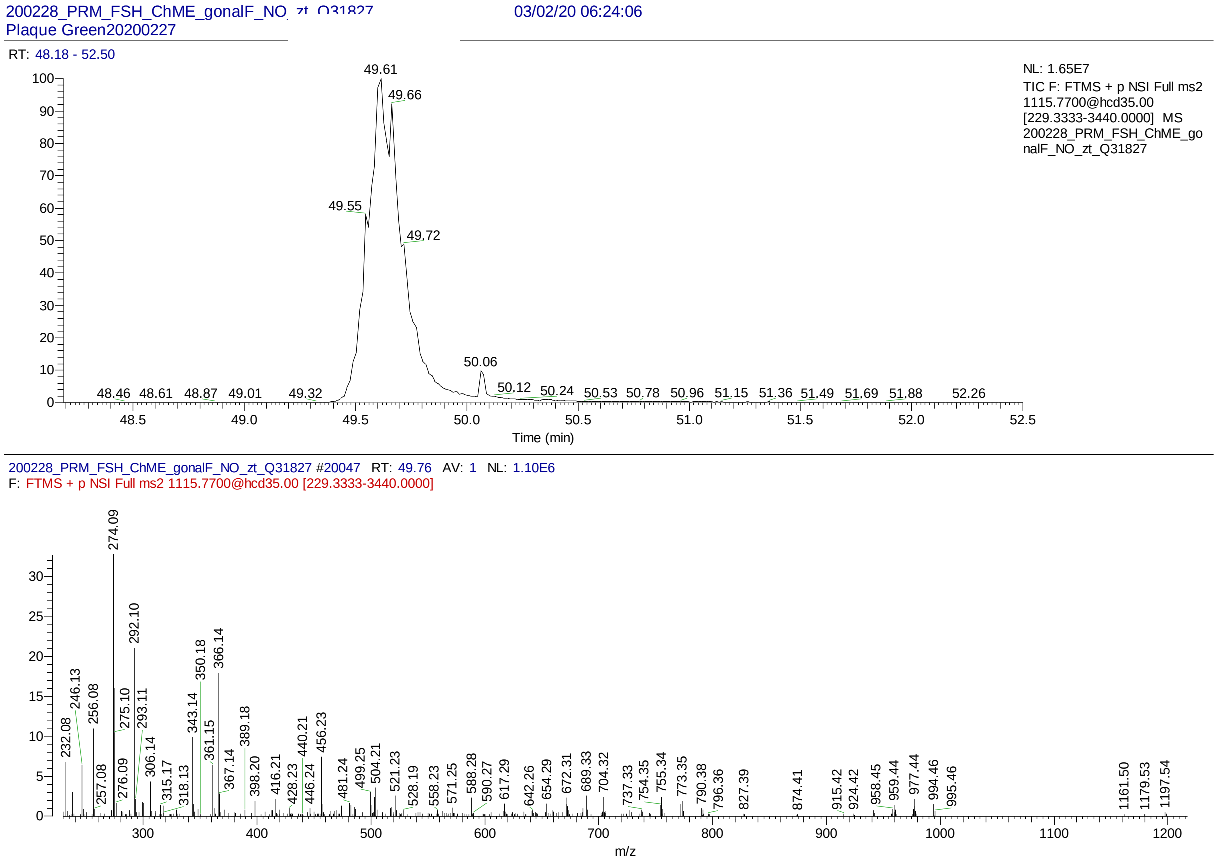

Figure S24. Glycopeptide #11 precursor ion XIC and relative MS2 spectrum.

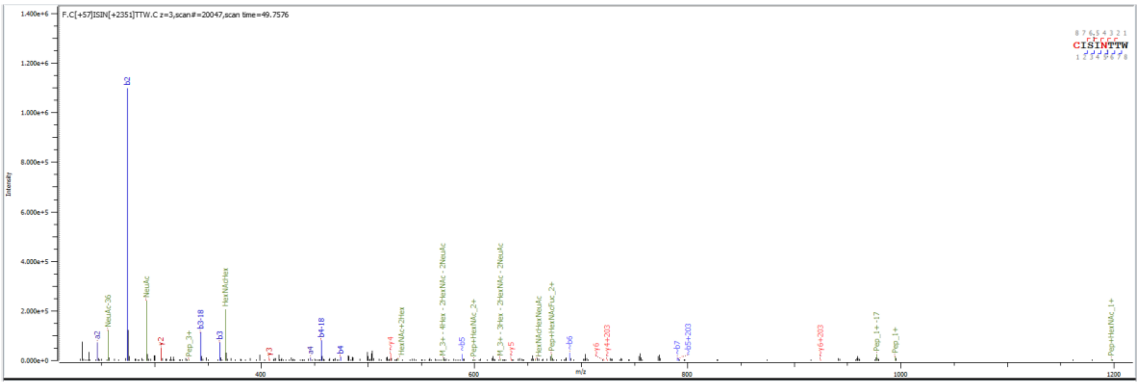

Figure S25. Glycopeptide #11 MS2 spectrum interpretation by Byonic software.

Glycopeptide #12

Table S20. Glycopeptide #12 information table. The highest fragment (*) was selected as quantifier.

FSHB_HUMAN Follitropin subunit beta							
**RT**	**Glycopeptide**	***N*-glycan site**	**Glycan**	**Precursor ion**	**z**	**Most intense Fragment ion**	**z**
49.34	F.CISIN[+2496.888]TTW.C	b Asn 24	F2A2G2S2	1746.173	2	274.09*	1
						292.08	1
						366.14	1

*selected as quantifier.

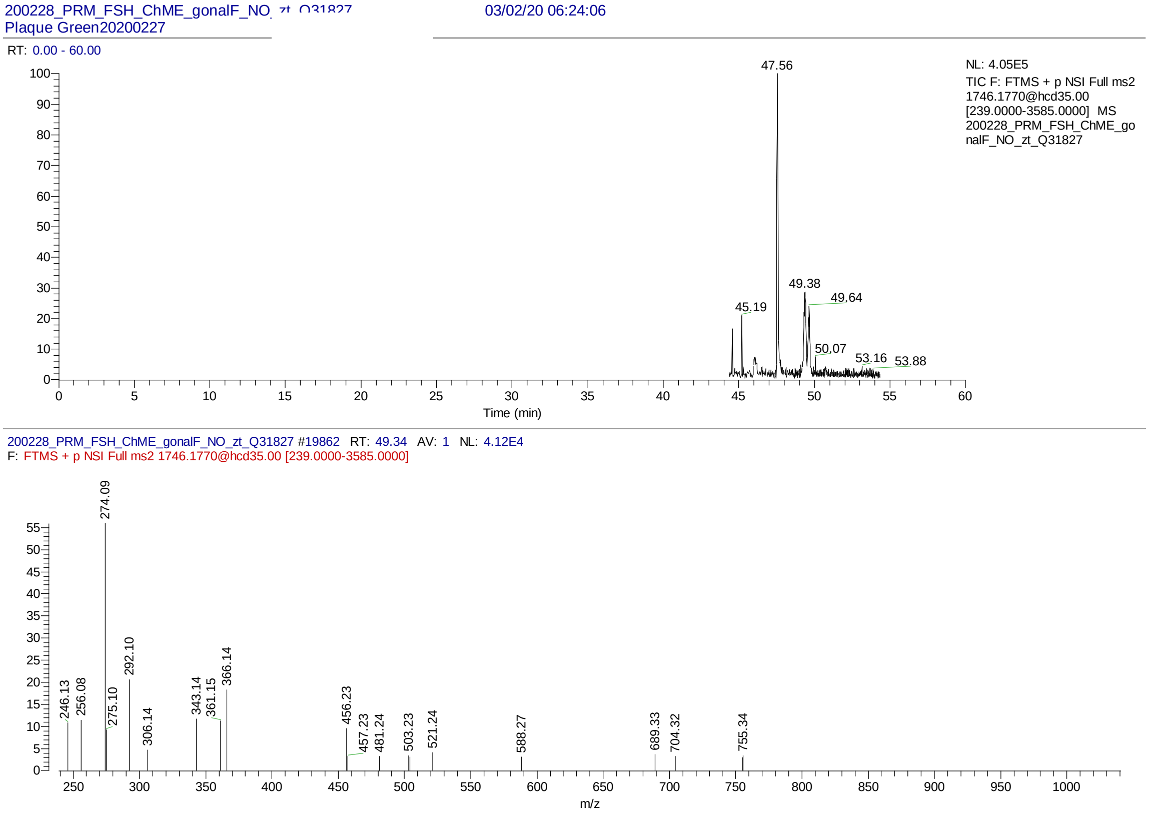

Figure S26. Glycopeptide #12 precursor ion XIC and relative MS2 spectrum.

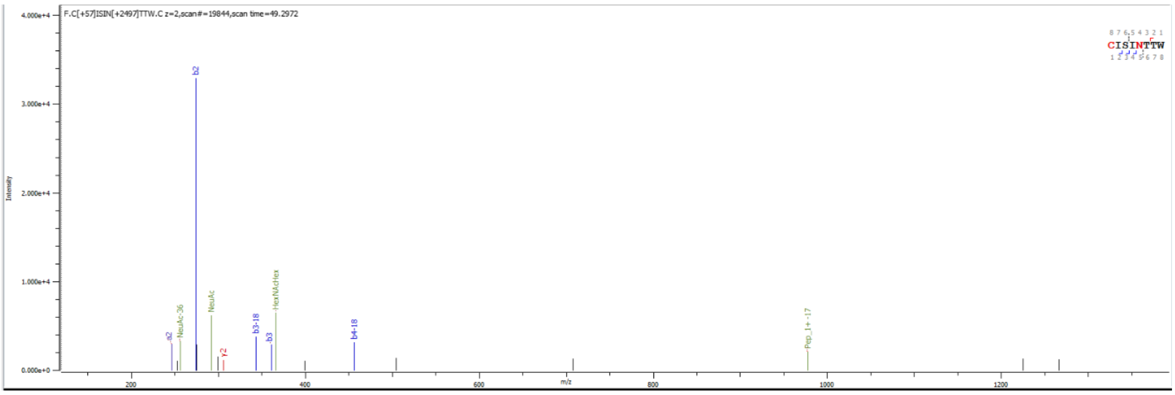

Figure S27. Glycopeptide #12 MS2 spectrum interpretation by Byonic software.

Glycopeptide #13

Table S21. Glycopeptide #13 information table. The highest fragment (*) was selected as quantifier.

FSHB_HUMAN Follitropin subunit beta							
**RT**	**Glycopeptide**	***N*-glycan site**	**Glycan**	**Precursor ion**	**z**	**Most intense Fragment ion**	z
44.72	F.CISIN[+2076.750]TTW.C	b Asn 24	F2A2G3	1536.109	2	274.12	1
						361.15	1
						366.14*	1

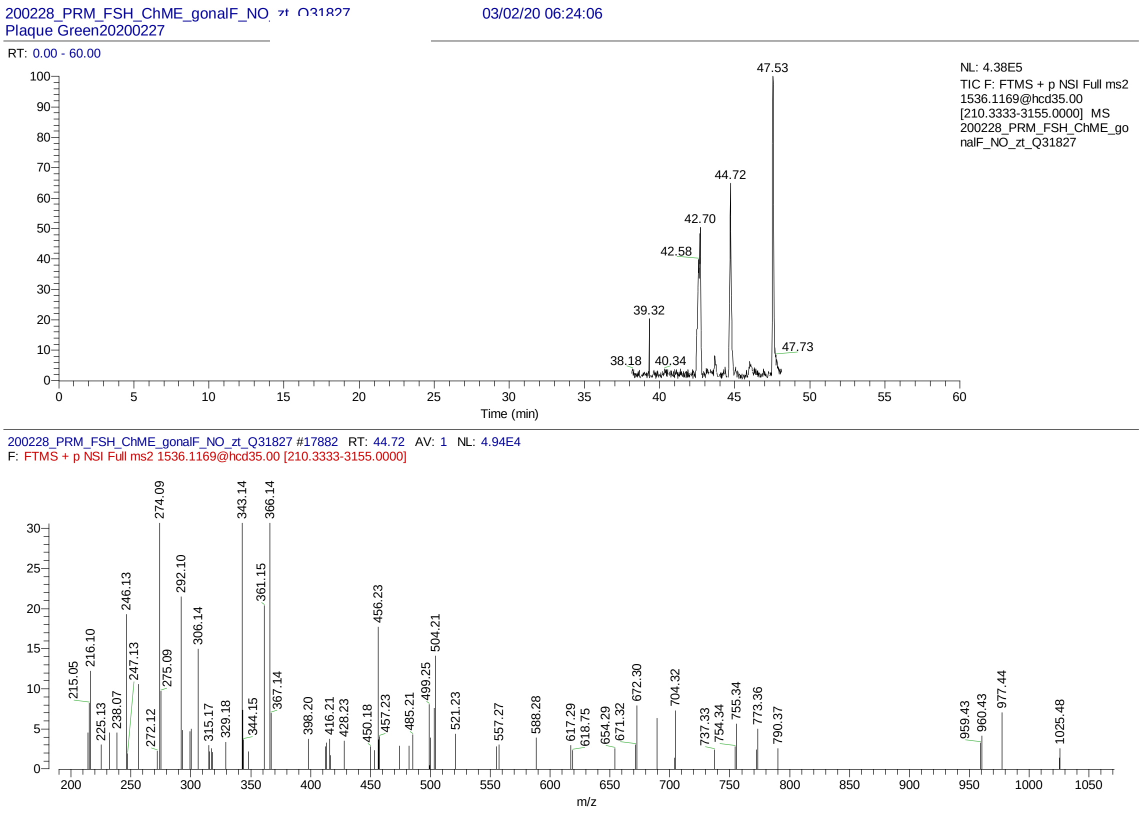

Figure S28. Glycopeptide #13 precursor ion XIC and relative MS2 spectrum.

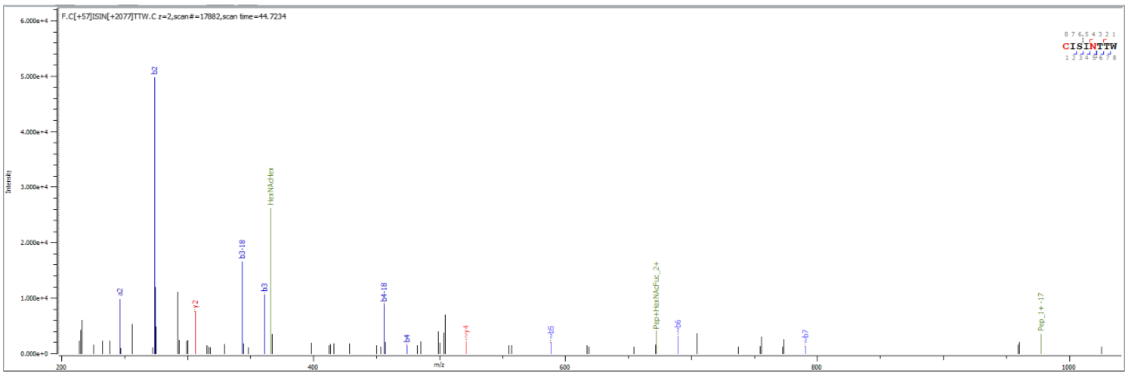

Figure S29. Glycopeptide #13 MS2 spectrum interpretation by Byonic software.

Glycopeptide #14

Table S22. Glycopeptide #14 information table. The highest fragment (*) was selected as quantifier.

FSHB_HUMAN Follitropin subunit beta							
**RT**	**Glycopeptide**	***N*-glycan site**	**Glycan**	**Precursor ion**	**z**	**Most intense Fragment ion**	**z**
						204.09*	1
36.97	F.CISIN[+1768.640]TTW.C	b Asn 24	FA2G2	921.706	3	366.14	1
						512.2	1

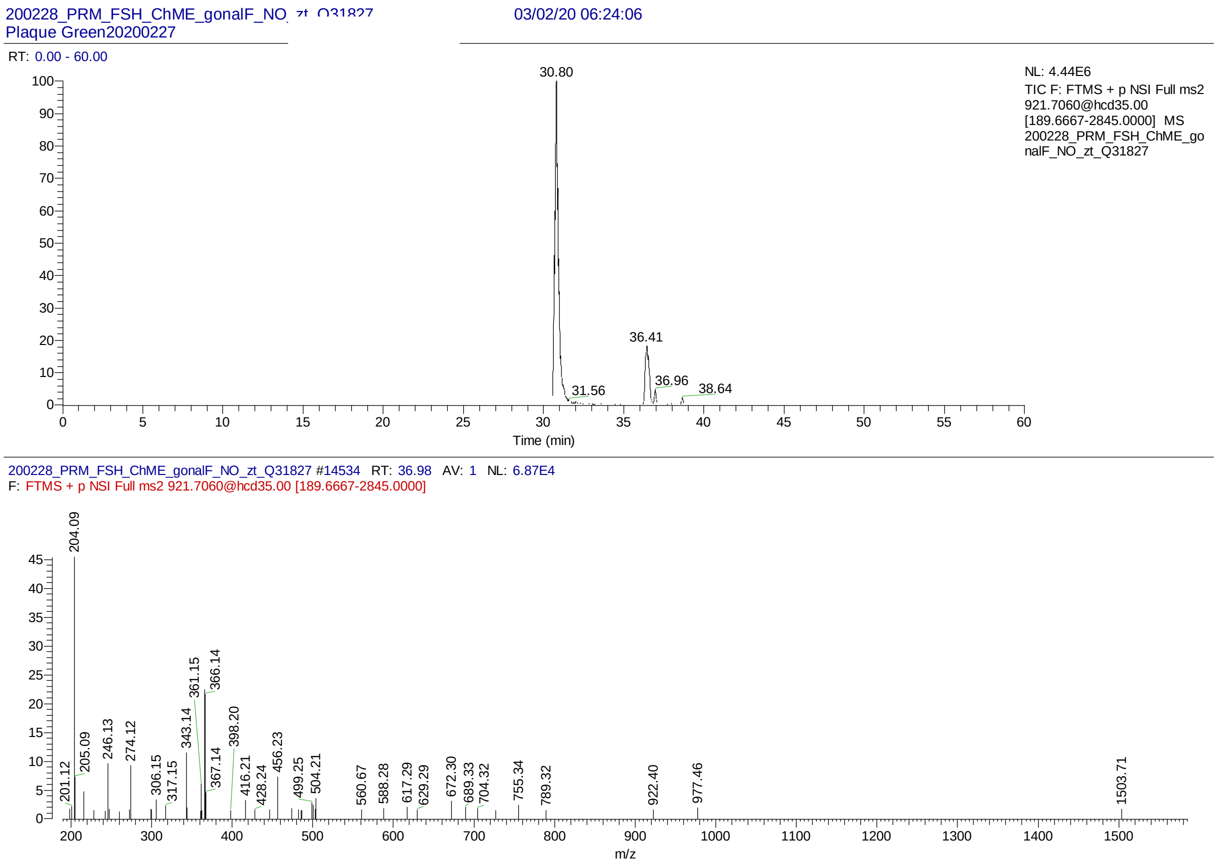

Figure S30. Glycopeptide #14 precursor ion XIC and relative MS2 spectrum.

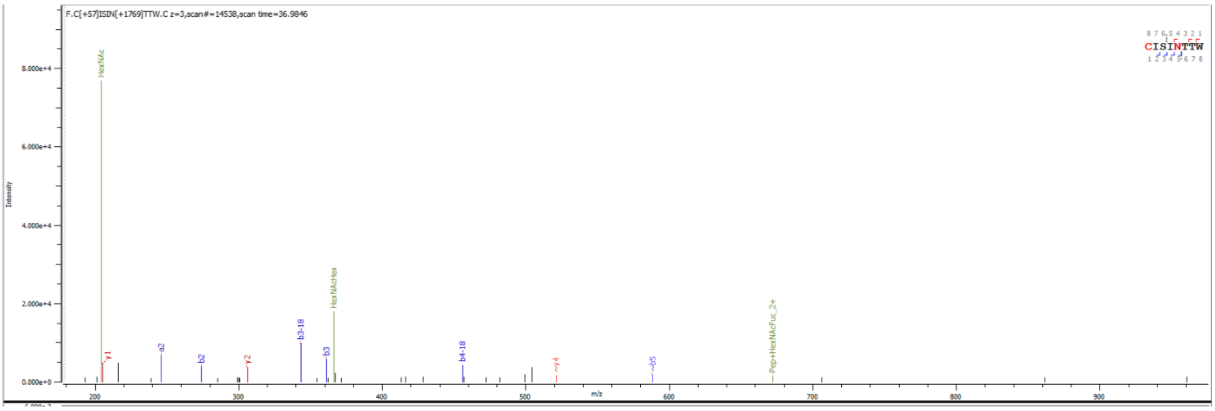

Figure S31. Glycopeptide #14 MS2 spectrum interpretation by Byonic software.

Glycopeptide #15

Table S23. Glycopeptide #15 information table. The highest fragment (*) was selected as quantifier.

FSHB_HUMAN Follitropin subunit beta							
RT	Glycopeptide	*N*-glycan site	Glycan	Precursor ion	z	Most intense Fragment ion	z
44.75	F.CISIN[+2059.735]TTW.C	b Asn 24	FA2G2S1	1527.603	2	274.09*	1
						292.08	1
						366.14	1
						671.31	2

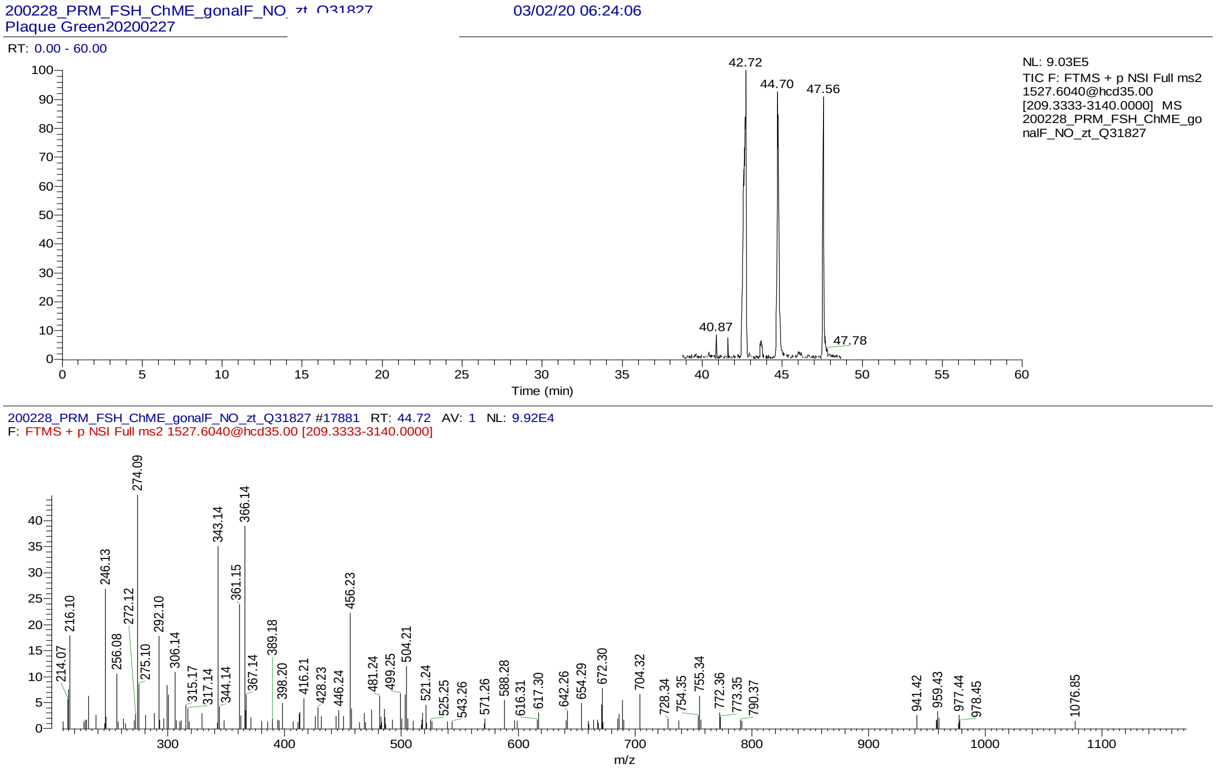

Figure S32. Glycopeptide #15 precursor ion XIC and relative MS2 spectrum.

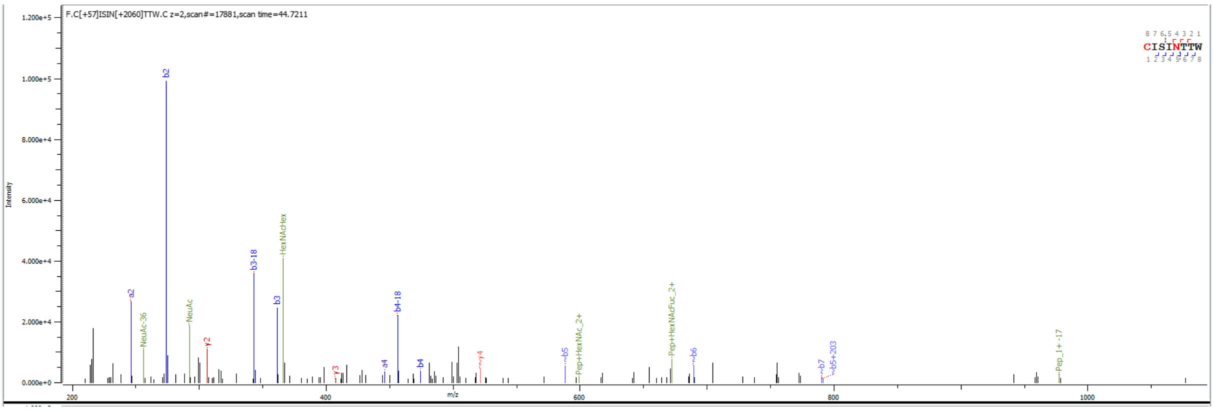

Figure S33. Glycopeptide #15 MS2 spectrum interpretation by Byonic software.
